# Transcriptome analysis of mammary epithelial subpopulations identifies novel determinants of lineage commitment and cell fate

**DOI:** 10.1186/1471-2164-9-591

**Published:** 2008-12-08

**Authors:** Howard Kendrick, Joseph L Regan, Fiona-Ann Magnay, Anita Grigoriadis, Costas Mitsopoulos, Marketa Zvelebil, Matthew J Smalley

**Affiliations:** 1 Breakthrough Breast Cancer Research Centre, The Institute of Cancer Research, 237 Fulham Road, London, SW3 6JB, UK; 2 Breakthrough Breast Cancer Research Unit, Guy's Hospital, London, SE1 9RT, UK

## Abstract

**Background:**

Understanding the molecular control of cell lineages and fate determination in complex tissues is key to not only understanding the developmental biology and cellular homeostasis of such tissues but also for our understanding and interpretation of the molecular pathology of diseases such as cancer. The prerequisite for such an understanding is detailed knowledge of the cell types that make up such tissues, including their comprehensive molecular characterisation. In the mammary epithelium, the bulk of the tissue is composed of three cell lineages, namely the basal/myoepithelial, luminal epithelial estrogen receptor positive and luminal epithelial estrogen receptor negative cells. However, a detailed molecular characterisation of the transcriptomic differences between these three populations has not been carried out.

**Results:**

A whole transcriptome analysis of basal/myoepithelial cells, luminal estrogen receptor negative cells and luminal estrogen receptor positive cells isolated from the virgin mouse mammary epithelium identified 861, 326 and 488 genes as highly differentially expressed in the three cell types, respectively. Network analysis of the transcriptomic data identified a subpopulation of luminal estrogen receptor negative cells with a novel potential role as non-professional immune cells. Analysis of the data for potential paracrine interacting factors showed that the basal/myoepithelial cells, remarkably, expressed over twice as many ligands and cell surface receptors as the other two populations combined. A number of transcriptional regulators were also identified that were differentially expressed between the cell lineages. One of these, *Sox6*, was specifically expressed in luminal estrogen receptor negative cells and functional assays confirmed that it maintained mammary epithelial cells in a differentiated luminal cell lineage.

**Conclusion:**

The mouse mammary epithelium is composed of three main cell types with distinct gene expression patterns. These suggest the existence of a novel functional cell type within the gland, that the basal/myoepithelial cells are key regulators of paracrine signalling and that there is a complex network of differentially expressed transcription factors controlling mammary epithelial cell fate. These data will form the basis for understanding not only cell fate determination and cellular homeostasis in the normal mammary epithelium but also the contribution of different mammary epithelial cell types to the etiology and molecular pathology of breast disease.

## Background

The function of complex tissues, such as the mammary epithelium, is a product of the interactions between their constituent cell types. In such tissues, disease states like cancer are essentially a failure of this cellular homeostasis and are characterised by insensitivity of cells to external regulatory factors and aberrant cell fate choices. Understanding the molecular regulation of the individual cell types in complex tissues is, therefore, a prerequisite for understanding disease states. Furthermore, advances in molecular pathology have demonstrated that different disease phenotypes correlate with different gene expression profiles [1]. In a complex tissue, composed of different cell types with different molecular characteristics, the gene expression profiles of different diseases may reflect the contribution of different cell types to that disease. Therefore, a detailed molecular characterisation of the cell types in a complex tissue is essential for the interpretation of the molecular pathology of its diseases.

The resting adult mammary epithelium consists of two main structures, alveoli (which develop into milk-secreting lobulo-alveolar structures upon pregnancy) and ducts (which carry the milk from the lobulo-alveolar structures to the nipple) [2]. These two structures are themselves composed of two main epithelial cell layers, basal cells and luminal cells. The basal cell layer mainly consists of myoepithelial cells which contract in response to oxytocin release during lactation to force milk down the ducts to the nipple. Recent work has demonstrated that the basal cell layer also contains the mammary epithelial stem cell compartment [3-7]. The luminal cell layer has been shown to be composed of two functionally distinct lineages defined by expression of the cell surface proteins CD24 and Sca-1. CD24^+/High ^Sca-1^+ ^luminal cells express estrogen receptor alpha (ER), as well as receptors for prolactin and progesterone (the luminal ER^+ ^compartment), while CD24^+/High ^Sca-1^- ^luminal cells (the luminal ER^- ^compartment) express genes (at low levels) for milk proteins even in the virgin and likely include alveolar progenitors [5,7-9].

Although it is known that the stem cells can generate all the myoepithelial, luminal ER^- ^and luminal ER^+ ^daughter cell types [5], the mechanisms which control cellular homeostasis, fate determination and lineage commitment in the mammary epithelium are poorly understood. They are likely to be a product of complex interactions between cell extrinsic paracrine influences, cell intrinsic transcriptional regulators and epigenomic modifications [10]. Some progress has been made towards understanding some of the cell intrinsic factors involved. For instance, Gata3 was recently identified as a transcription factor important in specifying commitment in the general luminal lineage [11,12] and Elf5 was shown to be a specifier of alveolar cell fate [13]. A number of the cell extrinsic (paracrine) factors operating within the mammary epithelium have also been characterised, such as Wnt-4, which acts downstream of progesterone signalling in ductal side-branching [14] and the EGF-family member Amphiregulin [15,16], which is produced by ER^+ ^cells in response to estrogen and stimulates mammary stem cell activity (most likely acting indirectly via non-epithelial cells and additional paracrine factors) [17]. The Notch signalling pathway has also been shown to be an important determinant of luminal cell fate [18,19]. However, the full extent and nature of paracrine interactions in the mammary gland, and the degree to which the different lineages contribute to them, and are defined by them, is still not fully understood.

Gene expression patterns have been previously examined in the mouse mammary gland, either as changes in gene expression across the whole tissue in developmental timecourses [20,21] or as comparisons between the total epithelium and the mammary stroma [12]. In one report, gene expression patterns were examined in mouse luminal and myoepithelial cells purified by flow sorting as well as in stem cell enriched basal cell populations [6]. However, these stem cell gene signatures were found to be not significantly different from the myoepithelial signatures, suggesting they were derived from cell populations dominated by myoepithelial cells. The purity of the basal stem cell populations remains a persistent problem due the difficulty of isolating pure (as opposed to enriched) stem cell fractions. A number of gene expression studies have also been carried out on human breast cells. The response of human breast epithelium to estrogen has been analysed at the gene expression level in breast cancer cell lines *in vitro *and as xenografts [22-24], in normal breast tissue maintained as xenografts [25] and in normal human ER^+ ^breast cells isolated by transduction of primary breast epithelial cells with a virus carrying an estrogen response element driving GFP expression [26]. The comparative gene expression profiles of normal human myoepithelial [27,28], basal non-myoepithelial (with a cell surface phenotype CD10^- ^CD44^+^) [29] and luminal epithelial cells [27-29] have also been examined, as have the gene expression profiles of different *in vitro *progenitor (colony-forming) subpopulations of normal breast epithelial cells [18]. However, to date no genome-wide transcriptome study has made a direct comparison between the two luminal epithelial populations (ER^- ^and ER^+^) and the basal/myoepithelial cells, confounding the molecular characterisation of the luminal cells and preventing the analysis of the lineage commitment of, and interactions between, the two luminal cell types and the other cell types in the gland.

The aim of this study, therefore, was to carry out the first comprehensive gene expression study which examined gene expression patterns in the three distinct mouse mammary epithelial populations, basal/myoepithelial, luminal ER^- ^and luminal ER^+^. The analysis was to concentrate on three specific areas. First, characterising cell-type specific patterns of gene expression which defined cell identity. Second, establishing a broad overview of the likely extent and nature of paracrine interactions amongst the populations. Last, defining cell intrinsic factors which may be important in determining lineage commitment and cell fate in the mammary epithelium. The results of these analyses have, first, identified a novel potential function for a subpopulation of mammary luminal epithelial cells as non-professional immune cells; second, provided information on the large number of paracrine interactions yet to be fully characterised in the mammary gland and the likely complexity of their interactions; third, identified population-specific transcription factors which may have a role in lineage determination and fate specification in the mammary epithelium. In particular, we have used *in vitro *and *in vivo *functional analyses to demonstrate that *Sox6 *is a determinant of luminal cell fate.

## Results

### Identification of population-specific gene expression patterns in the virgin mouse mammary epithelium

**Figure 1:**
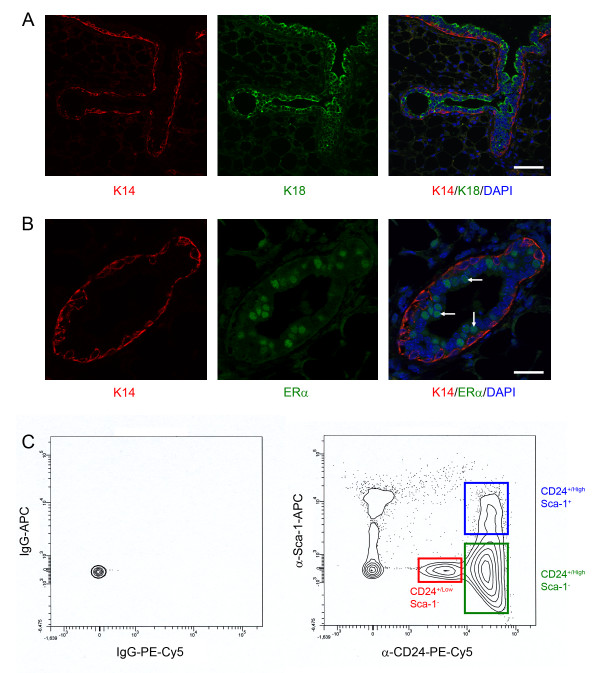
**CD24 and Sca-1 expression distinguishes basal, luminal ER^- ^and luminal ER^+ ^mammary epithelial cells**. A) Section through a branching duct in a mouse mammary gland stained for expression of keratin 14 in the basal myoepithelial layer (K14; red) and keratin 18 in the luminal epithelial layer (K18; green) and counterstained with DAPI (blue) to distinguish nuclei. Bar = 60 μm. B) Section through a mouse mammary duct stained for K14 (red) and ER (green) and counterstained with DAPI. Only a subset of luminal cell nuclei show ER staining (examples indicated by arrows). Bar = 25 μm. C) Flow cytometry plots of freshly isolated mouse mammary epithelial cells stained with control IgG (left) or anti-CD24 and anti-Sca-1 (right) antibodies. The regions corresponding to the CD24^+/Low ^Sca-1^- ^(red), CD24^+/High ^Sca-1^- ^(green) and CD24^+/High ^Sca-1^+ ^(blue) populations are indicated.

**Figure 2:**
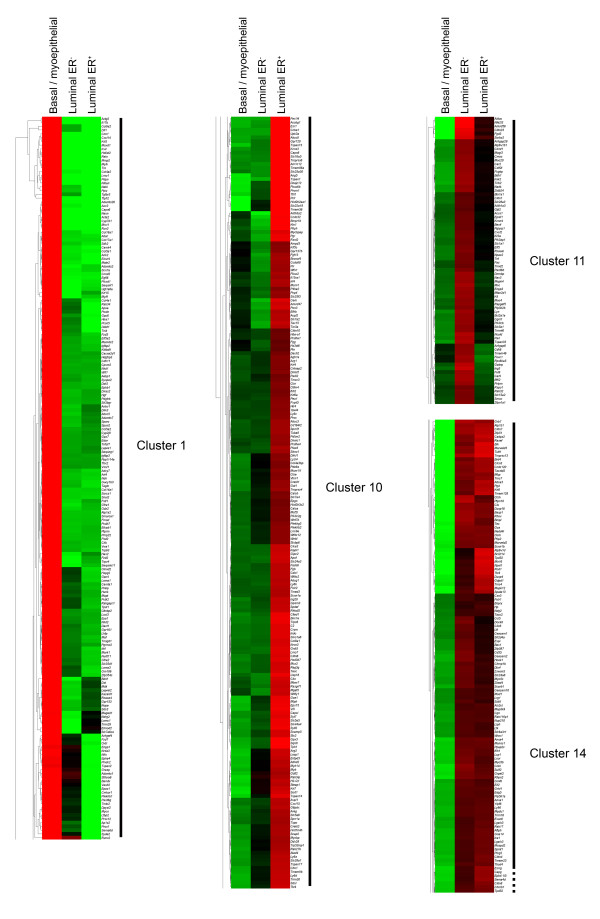
**Partial heat map and cluster analysis of differential gene expression across virgin mammary epithelial populations**. Clusters representative of the different gene expression patterns are shown. Red indicates high expression, green indicates low expression [see Additional file 3].

To carry out a comprehensive, whole genome gene expression analysis of the epithelial cell populations in the virgin mammary gland (Figure 1A and 1B), primary mouse mammary cells were isolated and stained with anti-CD24 and anti-Sca-1 antibodies. The CD24^+/Low ^Sca-1^-^, CD24^+/High ^Sca-1^- ^and CD24^+/High ^Sca-1^+ ^cells were separated by flow cytometry (Figure 1C) and used to prepare RNA. To demonstrate that these populations corresponded to basal/myoepithelial, luminal ER^- ^and luminal ER^+ ^cells respectively, as we have previously described [5], relative gene expression levels of the basal cell marker Keratin 14 (*Krt14*), the luminal cell marker Keratin 18 (*Krt18*) and Estrogen Receptor α (*Esr1*) were measured by quantitative real-time rtPCR (qPCR). The results showed that the CD24^+/Low ^Sca-1^- ^cells expressed *Krt14 *but not *Krt18 *and that the CD24^+/High ^Sca-1^- ^and CD24^+/High ^Sca-1^+ ^cells expressed *Krt18 *but not *Krt14*. They also showed that the CD24^+/High ^Sca-1^+ ^population was enriched for *Esr1 *expression compared to the other two populations. This agreed with previous data [5] and with staining of sections through the mouse mammary gland (Figure 1A and 1B) which showed that the basal cells were Keratin 14^+ ^(K14^+^), the luminal cells were K18^+ ^and that ER^+ ^cells were found exclusively in the K18^+ ^luminal cell layer. Therefore, these data confirmed the identity of the three populations [see Additional file 1] [5]. Next, a whole transcriptome gene expression analysis of the three populations was carried out using the Affymetrix platform (Mouse Expression Arrays MOE430 2.0). To identify genes whose expression in one population was different from its expression in the other two, Significance Analysis of Microarray (SAM) was applied [30,31]. Using this technique in a multiclass setting, the average gene expression within one group was compared with the average gene expression of all samples. Following repeated permutation of the data, the strength of the relationship between gene expression and its expressed group was established and a significance level determined. When a FDR cutoff of 0.0% was applied, 2182 probe sets [see Additional file 2] were identified which showed significant differential expression across the three populations. These 2182 probes corresponded to 1427 well known individual genes. Heat mapping and hierarchical cluster analysis of the genes identified 14 different gene clusters. The cluster analysis showed that some gene sets were characteristic of only one population (e.g. clusters 1, 10 and 11) whereas others were expressed in two of the three (e.g. cluster 14) (Figure 2) [see Additional file 3].

**Figure 3:**
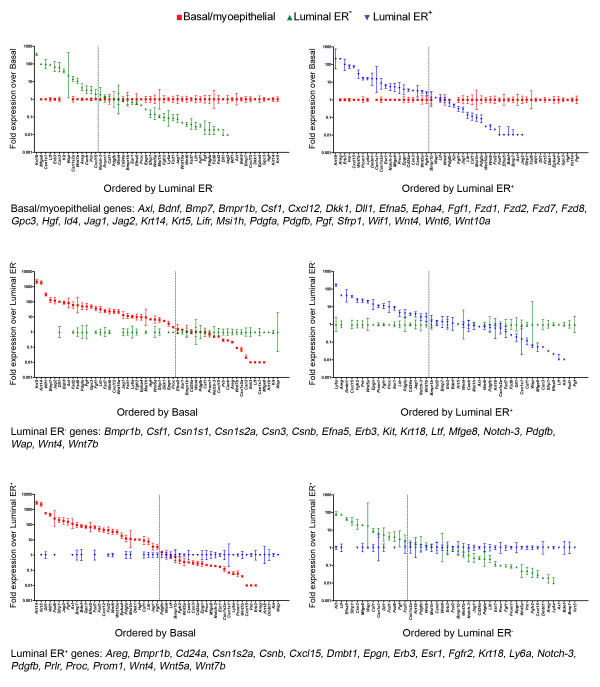
**qPCR analysis validates gene expression array analysis of virgin mammary epithelial cell gene expression**. Data from qPCR analysis of expression of 55 genes in triplicate independent samples of basal/myoepithelial cells, ER^- ^luminal cells and ER^+ ^luminal cells. Each data point is the mean level of expression, ± 95% confidence intervals, across the three samples of that population relative to the comparator sample. A 'round robin' comparison analysis was used as described in the Methods. Genes determined by this method to be characteristic of the comparator population are indicated below each pair of graphs.

To identify genes whose expression characterised the three subpopulations, the list of genes was split into three sets on the basis of relative abundance of expression. Any gene with a relative abundance of 2 or higher in a population was considered as population-specific. If a gene was represented by more than one probe set, the average of all probe sets was used for further analysis. This analysis identified 861, 326 and 488 genes as characteristic of basal/myoepithelial cells, luminal ER^- ^cells and luminal ER^+ ^cells, respectively [see Additional files 4, 5, 6]. To confirm our approach, a subset of genes specific for each of the three populations, as well as some which were common to two of the populations, were selected for qPCR validation. Furthermore, a number of genes previously shown to be relevant in mammary biology were included in this analysis, as was the data collected on *Krt14*, *Krt18 *and *Esr1 *expression in the populations [see Additional file 1], giving 58 genes in total (Figure 3). The qPCR probes for 3 genes failed to amplify any product in any of the populations. For a further 10 genes, there was no differential expression pattern in the array analysis. Technical problems, such as poor signal strength from the Affymetrix probe, cannot be ruled out in these cases. Of the remaining 45 genes which showed a differential expression pattern in both the array and qPCR analyses, 40 genes (88.9%) showed identical expression patterns across the three populations in both sets of data. With only 5 genes (11.1%) was the pattern of differential expression suggested by the array data different to that suggested by the qPCR analysis [see Additional file 7]. Overall, therefore, the qPCR data showed that the gene expression array dataset could be relied upon for a global picture of the biology of the three populations.

### Comparisons with previously published datasets

We compared our data with previous studies which have used a cell separation approach to isolate mouse [6] or human [27-29] mammary epithelial basal/myoepithelial and luminal subpopulations for gene expression profiling, to identify genes found in common between these datasets and our own data [see Additional files 8, 9, 10]. There was good concordance between the genes identified in our data and that of Stingl and colleagues [6] in their study of separated mouse epithelium. They identified 128 probes, corresponding to 80 well-annotated genes, significantly upregulated in their myoepithelial/mammary stem cell enriched cell fraction compared to their mammary colony forming cells (corresponding to the luminal cell fraction). Of these 80 genes, 49 (61%) were significantly enriched in our basal/myoepithelial cell dataset, three were enriched in both the basal/myoepithelial cells and the luminal ER^+ ^cells and two were enriched in both the basal/myoepithelial cells and the luminal ER^- ^cells [see Additional files 8 and 9]. Conversely, Stingl and colleagues identified 102 probes, corresponding to 66 well-annotated genes, significantly downregulated in their myoepithelial/mammary stem cell enriched cell fraction compared to their mammary colony forming cells. Of these, which would correspond to genes enriched in the luminal epithelial fraction, none were enriched in our basal/myoepithelial population but 28 (42%) were enriched in both our luminal populations, five were enriched only in the luminal ER^- ^cells and four only in the luminal ER^+ ^cells [see Additional files 8 and 10]. However, correspondence of our data with previously published datasets from separated human cells [27-29] was lower, although it tended to be better between the mouse myoepithelial and human myoepithelial data sets than between the luminal data sets from the different species [see Additional files 8, 9, 10].

### Interaction mapping of genes differentially expressed in mammary epithelial subpopulations identifies key processes and novel functions

To get a better understanding of the key biological processes occurring in each of the three cell types, we generated network interaction maps for the differentially expressed genes [see Additional files 11, 12, 13]. Interaction data derived from studies on human orthologues of the genes identified were used to create the network maps, as there is not enough data currently available purely from studies of mouse genes to make such an analysis meaningful.

When a basal/myoepithelial interaction map was constructed using both the differentially expressed genes and genes interpolated by the network mapping program (which allow connections to be extended and elaborated), the resulting network was extraordinarily complex (data not shown). For ease of interpretation, therefore, a basal/myoepithelial network was constructed using only direct interactions between genes characteristic of this cell population, with no interpolations [see Additional file 11]. This network identified two major interaction 'modules' and three minor ones. Such interaction modules are indicative of important processes occurring within a cell, as they are composed of cell-type specific genes and defined by multiple interactions between those genes. The two major interaction modules can be broadly characterised as an extracellular matrix module including multiple collagen genes and a cytoskeletal module including genes for the keratins, vimentin, and genes whose protein products are involved in regulation of cell shape, movement and contractility, such as the actin binding proteins MYH10 [32] and TPM2 [33] and smooth muscle gamma actin ACTG2 [34]. The minor modules indicated that the basal/myoepithelial cell population also has important processes based around Platelet-Derived Growth Factor (PDGF), Ephrin and Insulin-Like Growth Factor (IGF1) signalling. Ephrins are mediators of contact-dependent communication between cells [35] whereas both PDGF and IGF1 signalling are involved in paracrine cell-cell communication [36,37].

The luminal interaction maps were built using both cell-specific genes and interpolated genes [see Additional files 12 and 13]. As a result, it was less straightforward to define cell-specific interaction modules which would indicate the key cellular processes occurring in these cell types. We therefore developed a mathematical approach to defining the modules which required the assignment of network hubs, node clusters and contiguous differentially expressed network paths. To define network hubs (nodes having multiple interactions), nodes were ranked by descending connectivity. The minimum node connectivity in the top 10% of nodes was five for either network and this was therefore set as a threshold for identifying hubs. Differentially expressed hubs for the luminal ER^- ^and ER^+ ^networks are listed in Tables 1 and 2 respectively. There was limited overlap between the luminal ER^- ^and ER^+ ^network hubs with only three differentially expressed hubs being shared (*ERBB3*, *KRT18 *and *CD82*). The majority of hubs in both networks had a high content of physical interactions with the exception of *TNF*, *SP1*, *FAS*, *NFKB1 CREB1*, *EGR1 *and *SPI1*, which are almost exclusively transcriptional hubs. In the luminal ER^- ^network *TLR4*, *LY96*, *ERBB3*, *MUC1 *and *CD82 *were differentially expressed hubs with significant clustering character, although the strongest clustering was seen for the non-differentially expressed hubs *EGFR *and *ERBB2*. Clustering was less pronounced in the luminal ER^+ ^network with *PGR*, *ERBB3*, *FGG*, *FGB*, *CD82 *and *COL8A1 *forming significantly clustered differentially expressed hubs. With the exception of *TLR4 *and *LY96 *in the luminal ER^- ^network, clustering and connectivity were inversely correlated, with high connectivity nodes such as *ESR*, *PTN*, *CCL5*, *TNF *and *BCL2*, exhibiting very little or no clustering.

**Table 1:** Hubs and clustered nodes in the luminal ER^- ^network.

**Hub**	**k_node_**	**C_node_**	**Differentially expressed neighbours**	**Physical interaction content**	**Transcriptional interaction content**
CCL5*	17	0.000	0	35%	65%
TLR4*	17	**0.103**	2	**82%**	18%
TNF*	15	0.000	0	13%	**87%**
BCL2*	14	0.000	2	60%	40%
LY96*	14	**0.154**	2	**100%**	0%
KIT*	13	0.013	3	**92%**	8%
CCL2*	10	0.000	0	40%	60%
LYN*	10	0.022	1	**100%**	0%
ERBB3*	9	**0.194**	3	**100%**	0%
**SP1**	9	0.000	8	0%	**100%**
FAS*	8	0.000	0	25%	**75%**
MUC1*	8	**0.143**	1	**100%**	0%
**GRB2**	7	0.000	6	**100%**	0%
**UBQLN4**	7	0.000	6	**100%**	0%
CD82*	6	**0.133**	1	**100%**	0%
KRT18*	6	0.067	1	**83%**	17%
**NFKB1**	6	0.000	5	0%	**100%**
**ATXN1**	5	0.000	4	**100%**	0%
CD14*	5	**0.100**	2	40%	60%
**CREB1**	5	0.000	4	20%	**80%**
**EGFR**	5	**0.300**	4	**100%**	0%
**ERBB2**	5	**0.300**	4	**100%**	0%
**MAPK1**	5	0.000	4	**100%**	0%
**PIK3R1**	5	0.000	4	**100%**	0%
RPS6KA5*	5	0.000	0	**100%**	0%
**SPI1**	5	0.000	4	0%	**100%**

*Differentially-expressed genes. K_node_, node connectivity, C_node_, node clustering coefficient, calculated as C_node _= 2 n_node_/(k_node _(k_node _- 1)) where n_node _is the number of interactions between the hub first neighbours. Hubs exhibiting clustering are shown in bold. High content of physical or transcriptional interactions in the hub is shown in bold.

**Table 2:** Hubs and clustered nodes in the luminal ER^+ ^network.

**Hub**	**k_node_**	**C_node_**	**Differentially expressed neighbours**	**Physical interaction content**	**Transcriptional interaction content**
ESR1*	27	0.020	3	53%	47%
PTN*	19	0.012	6	**95%**	5%
TGFB1*	17	0.000	0	47%	53%
HIST1H4H*	16	0.000	0	**100%**	0%
HIST1H4I*	16	0.000	0	**100%**	0%
GADD45G*	13	0.051	2	**88%**	12%
EFEMP2*	10	0.022	1	**100%**	0%
LNX1*	10	0.000	0	**100%**	0%
AREG*	9	0.000	0	44%	56%
CAV1*	9	0.000	0	**78%**	22%
**EGFR**	9	0.056	6	**92%**	8%
CRELD1*	8	0.000	1	**100%**	0%
KRT18*	8	0.036	1	**89%**	11%
PGR*	8	**0.143**	1	63%	37%
**SP1**	8	0.036	6	0%	**100%**
EPS15*	7	0.000	0	**100%**	0%
ERBB3*	7	**0.143**	2	**100%**	0%
FGG*	7	**0.238**	1	**100%**	0%
PCDHA4*	7	0.000	0	**100%**	0%
**UBQLN4**	7	0.048	3	**100%**	0%
FGB*	6	**0.333**	1	**100%**	0%
**ATXN1**	5	0.000	1	**100%**	0%
CD82*	5	**0.200**	1	**100%**	0%
**CEBPB**	5	0.000	1	20%	80%
COL8A1*	5	**0.100**	1	**100%**	0%
**EGR1**	5	0.000	1	0%	**100%**
**ERBB2**	5	**0.200**	3	**100%**	0%
MBP*	5	0.000	0	**100%**	0%
MLLT4*	5	0.000	2	**100%**	0%
**SMAD2**	5	0.000	2	**100%**	0%
**TP53**	5	0.000	2	**80%**	20%

See legend to Table 1 for details. *Differentially expressed genes.

To identify modules within the networks, a three-pass approach was adopted [see Additional file 14]. Initially all differentially expressed hubs were identified and, where direct links existed between them in the network, these were used to provide the backbone for each module. In the second pass, modules were allowed to expand by the addition of differentially expressed nodes with lower connectivity (such that they did not qualify as hubs), if they were directly linked to differentially expressed hubs. In the third pass, non-differentially expressed hubs were incorporated, if they were connecting at least two differentially expressed hubs. This step allowed for bridging between modules, providing further information about their proximity and global topology within the luminal ER^- ^and luminal ER^+ ^networks [see Additional files 15 and 16].

Following the initial two passes, four distinct modules were established for the luminal ER^- ^network [see Additional files 14 and 15]: the TLR (nodes *TLR4*, *LY96 *and *CD14*), KIT (nodes *KIT*, *ERBB3*, *LYN*, *TEC*, *GRB7*, *MUC1 *and *CD82*), KRT (nodes *KRT18 *and *KRT8*) and BCL2 (nodes *BCL2*, *BIK *and *BNIPL*) modules (Table 3). Nodes *TNF*, *FAS*, *CCL5*, *CCL2 *and *RPS6KA5 *were integrated in the BCL2 subnet only at the third pass through a network of transcriptional interactions, forming the TNF/FAS module [see Additional file 14]. In addition, the KRT module merged into the KIT module at this stage. The TLR and KIT modules contained predominantly physical interactions whereas the TNF/FAS module displayed a very strong transcriptional character (82%). The three modules exhibit topological proximity, defining a single subnetwork with 39 connections and 27 nodes [see Additional files 14 and 15]. Compared to the luminal ER^- ^network overall, this subnetwork showed higher clustering and, whilst maintaining the average network connectivity, it exhibited a significantly shorter mean shortest path and a very low power exponent. Removing this subnetwork from the overall luminal ER^- ^network yielded a highly fragmented graph ('non-module subnetwork') with no clustering and very low connectivity [see Additional file 17].

**Table 3:** Summary of modules from luminal networks

**Module**	**Constituent hubs**	**Function**	**Ref.**	**Inferred module function**
**Luminal ER**^-^				
**TLR**	CD14	Component of LPS receptor	[38]	Passive immune
	LY96	Component of LPS receptor	[38]	response
	TLR4	Component of LPS receptor	[38]	
				
**KIT/KRT**	CD82	Tetraspanin	[86]	Kinase signalling
	ERBB3	ERBB family tyrosine kinase	[51]	pathways and
	GRB7	Signalling adaptor protein	[87]	cellular structural
	KIT	Receptor tyrosine kinase	[88]	integrity
	KRT8	Cytoskeletal component	[89]	
	KRT18	Cytoskeletal component	[89]	
	LYN	Src family kinase	[90]	
	MUC1	Mucin family cell surface protein	[91]	
	TEC	Non-receptor tyrosine kinase	[92]	
				
**TNF/FAS**	BCL2	Anti-apoptotic protein	[93]	Inflammatory
	BIK	Pro-apoptotic protein	[94]	response and cell
	BNIPL	Putative pro-apoptotic protein	[95]	death
	CCL2	Inflammatory chemokine	[96]	
	CCL5	Inflammatory chemokine	[96]	
	FAS	Prototype death receptor	[97]	
	RPS6KA5	Serine/threonine kinase; controls transcriptional response to TNF	[98]	
	TNF	Pro-inflammatory cytokine	[97]	
				
**Luminal ER**^+^				
**ESR**	CRELD1	Cell adhesion molecule	[99]	Multifunctional
	ESR1	Transcriptional regulator	[56]	
	GADD45G	DNA damage response	[100]	
	GIPC2	Mediate cross-talk between cell signalling pathways	[101]	
	KRT19	Cytoskeletal component	[89]	
	MYCBP	Interacts with Myc and A-kinase anchoring proteins	[102]	
	PGR	Transcriptional regulator	[39]	
	PTN	Activation of stromal fibroblasts and stimulation of angiogenesis	[103]	
	STC2	Secreted glycoprotein with possible autocrine or paracrine function	[104]	
	WFDC2	Secreted anti-microbial protein	[105]	
				
**ERBB3**	CD82	Tetraspanin	[86]	Signal transduction
	ERBB3	ERBB family tyrosine kinase	[51]	
	GRB7	Signalling adaptor protein	[87]	
				
**MLLT4**	EPHB6	Cell-cell interactions/adhesion	[35]	Cell adhesion
	MLLT4	Cell adhesion	[106]	
	NRXN4	Cell-cell interactions/adhesion	[107]	
				
**COL8A1**	COL8A1	Extracellular matrix protein	[108]	Generation of
	EFEMP2	Extracellular matrix protein	[109]	extracellular matrix
				
**KRT**	KRT8	Cytoskeletal component	[89]	Cellular structural
	KRT18	Cytoskeletal component	[89]	integrity
				
**FGG**	FGB	Extracellular matrix protein	[110]	Generation of
	FGG	Extracellular matrix protein	[110]	extracellular matrix

For the luminal ER^+ ^network, six distinct modules were observed following the initial two passes [see Additional files 14 and 16]: the ESR (nodes *ESR1*, *PGR, GADD45G*, *PTN*, *CRELD1*, *GIPC2*, *KRT19*, *MYCBP*, *STC2 *and *WFDC2*), ERBB3 (nodes *ERBB3*, *CD82 *and *GRB7*), MLLT4 (nodes *MLLT4*, *EPHB6 *and *NRXN3*), COL8A1 (nodes *COL8A1 *and *EFEMP2*), KRT (nodes *KRT18 *and *KRT8*) and FGG (nodes FGG and FGB) modules (Table 3). After the third pass, modules ESR, ERBB3, KRT, COL8A1 and MLLT4 were merged into a large subnetwork with differentially expressed hubs *TGFB1*, *AREG*, *MBP*, *CAV1*, *EPS15 *and *PCDHA4 *playing an interconnecting role [see Additional file 14]. Unlike the luminal ER^- ^network, where all modules were proximal to each other, more fragmentation was observed for the luminal ER^+ ^network, with module FGG and differentially expressed hubs *HIST1H4H*, *HIST1H4I *and *LNX1 *not interconnected with the module subnetwork via any other hubs. The luminal ER^+ ^subnetwork also had a much stronger physical interaction character than the luminal ER^- ^subnetwork. Compared to the overall luminal ER^+ ^network, its subnetwork showed high clustering and, whilst maintaining the average network connectivity, exhibited a significantly shorter mean shortest path. As with the luminal ER^- ^subnetwork, removing the luminal ER^+ ^subnetwork from the overall luminal ER^+ ^network gave a highly fragmented graph with no clustering and very low connectivity [see Additional file 17]. The luminal ER^- ^and luminal ER^+ ^modules, their component nodes and potential functions are summarised in Table 3. They suggest that the major processes occurring in the luminal ER^- ^cells are tyrosine kinase cell signalling pathways in association with cellular cytoskeletal components (the keratins) as well as passive immune signalling and inflammatory response processes. The picture in the luminal ER^+ ^cells is less straightforward. There is no obvious unifying theme to the major ESR module, which leaves five minor modules involved in signal transduction, the cytoskeleton, cell adhesion and maintaining the extracellular matrix (although compared to the basal/myoepithelial cells, the role of the luminal ER^+ ^cells in this is minor).

**Figure 4:**
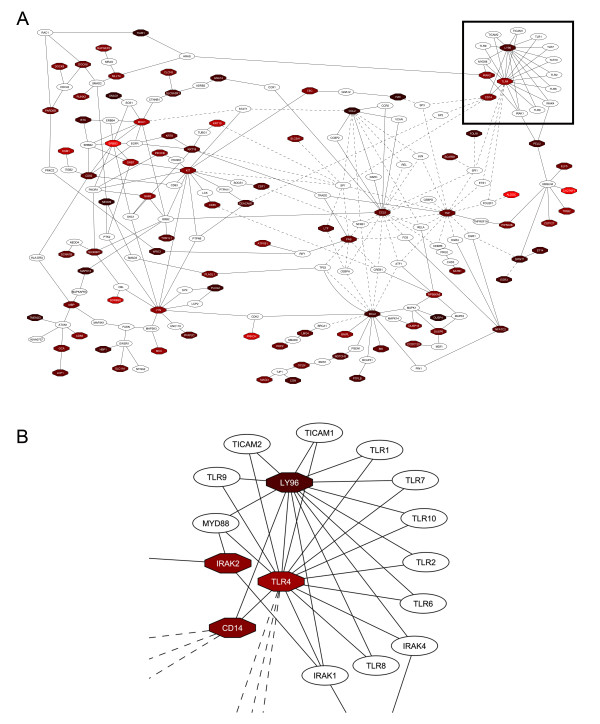
**Network interaction map for luminal ER^- ^specific genes**. Interaction data based on physical interactions (solid lines) and transcriptional interactions (dashed lines). The nodes are colour coded to indicate relative strengths of expression of the gene within the cell population. Brighter reds indicate highest levels of expression. Darker reds indicate genes less strongly expressed (although still with enriched expression within the population compared to the other cell types). White nodes indicate interpolated genes used by the network mapping software to extend and link the network [see Additional file 12]. A) Whole interaction map. Box indicates Toll-like receptor module. B) Enlargement of Toll-like receptor interaction module.

**Figure 5:**
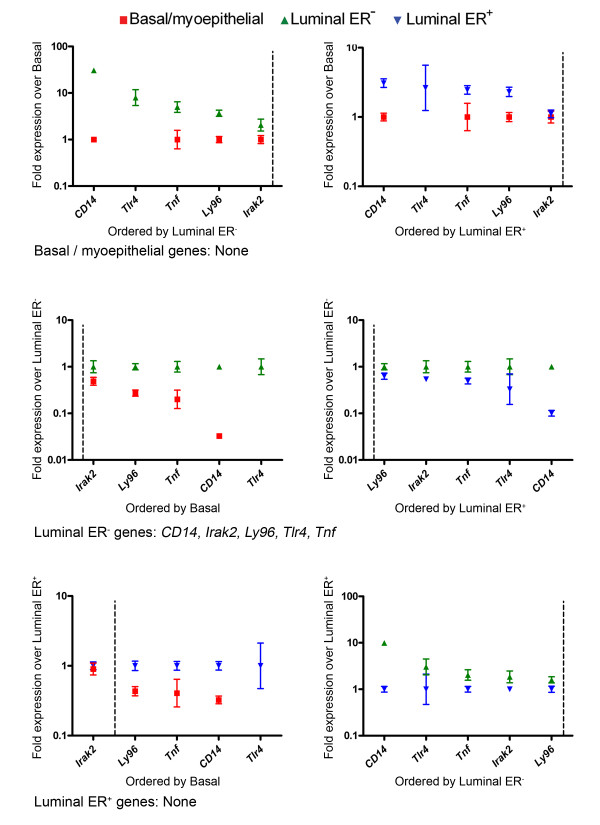
**qPCR analysis confirms that luminal ER^- ^cells are enriched for expression of LPS receptor pathway genes**. Data from qPCR analysis of *CD14*, *Irak2*, *Ly94*, *Tlr4 *and *Tnf *gene expression in triplicate independent samples of basal/myoepithelial cells, luminal ER^- ^cells and luminal ER^+ ^cells. Each data point is the mean level of expression, ± 95% confidence intervals, across the three samples of that population relative to the comparator sample. The 'round robin' comparison method and assignment of genes to the populations were carried out as described in the Methods.

The TLR module within the luminal ER^- ^network was of particular interest (Figure 4) [38], as it indicated a novel role of these cells as non-professional immune cells which can respond to bacterial lipopolysaccharide. To confirm that the luminal ER^- ^population was indeed enriched for cells expressing components of the CD14-TLR4 signalling complex, qPCR analysis of relative expression levels of the three components of the LPS receptor, *CD14*, *Ly96 *and *Tlr4*, its downstream effecter *Irak2 *and the LPS receptor responsive gene *Tnf *were carried out [38]. The results (Figure 5) confirmed that all five genes were specifically enriched in the luminal ER^- ^cells compared to both the basal/myoepithelial and luminal ER^+ ^cells. In particular, *CD14*, *Tlr4 *and *Tnf *were expressed at 10-fold, 3-fold and 2-fold, respectively, higher levels in the luminal ER^- ^cells compared to the luminal ER^+ ^cells. *CD14 *and *Tnf *were expressed at 30-fold and 5-fold higher levels in the luminal ER^- ^cells compared to the basal/myoepithelial cells. *Tlr4 *expression was undetectable in the basal/myoepithelial cells.

**Figure 6:**
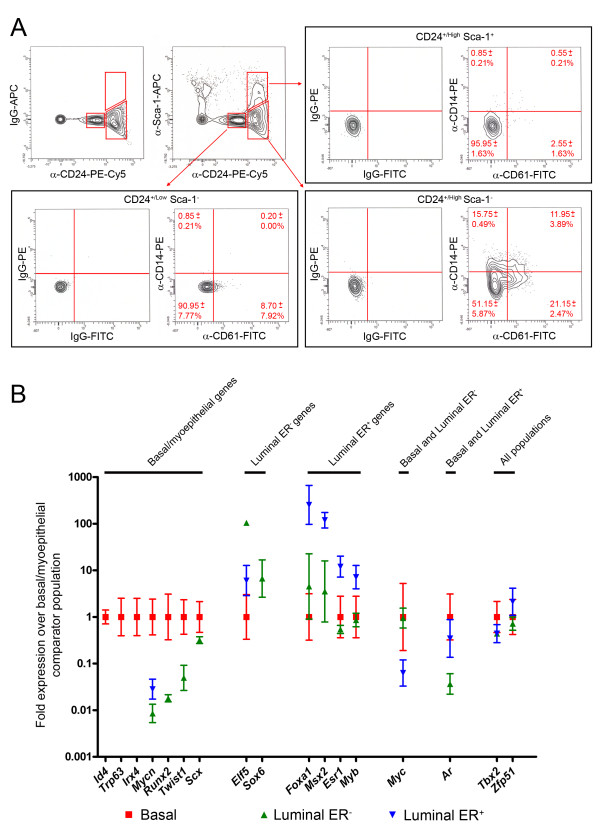
**Identification of CD14^+ ^and CD61^+ ^cells in the luminal ER^- ^population and confirmation of differential expression of transcriptional regulators**. A) Flow cytometric analysis of virgin mouse mammary cells stained with anti-CD24 and a control IgG or anti-CD24 and anti-Sca-1, to define the myoepithelial/basal, luminal ER^- ^and luminal ER^+ ^populations. The staining patterns of the three populations with control IgGs or anti-CD14 and anti-CD61 is indicated. The percentages in the CD14/CD61 quadrants indicate the mean percentage (± SD; n = three independent sorts) of that epithelial subpopulation (not of the total epithelium) falling into that quadrant. B) qPCR analysis of expression of transcriptional regulators in mammary epithelial subpopulations. Mean gene expression levels ± 95% confidence limits for the seventeen transcriptional factors indicated on the X-axis are given relative to the basal/myoepithelial population. The genes are grouped according to the cell population(s) which has the strongest level of expression. If there is no data point for a population for a given gene, then that gene could not be detected in that population.

To determine whether CD14 expression, and thus the potential to be able to respond to LPS, was a general property of all luminal ER^- ^cells or only a subfraction, freshly isolated primary cells were stained with anti-CD24 and anti-Sca-1 antibodies, to enable the three main cell compartments to be identified, as well as with anti-CD14 and anti-CD61 (proposed as a marker of progenitor cells) [11] antibodies. The results (Figure 6A) showed that the CD24^+/High ^Sca-1^- ^luminal ER^- ^population is itself composed of four different subpopulations, namely CD14^- ^CD61^- ^cells, CD14^+ ^cells, CD61^+ ^cells and CD14^+ ^CD61^+ ^cells. Interestingly, a small number of CD24^+/Low ^Sca1^- ^basal cells also showed elevated levels of CD61 expression.

### The luminal ER^+ ^gene expression pattern is not an 'estrogen-responsive' gene expression signature

Upon examination of the luminal ER^+ ^gene network, we noted that few of the genes enriched in the luminal ER^+ ^population were directly linked to *ESR1 *by transcriptional interactions, with the exception of the progesterone receptor (*PGR*) [39] and the cytoskeletal protein keratin 19 (*KRT19*) [40]. This suggested that the gene signature of the luminal ER^+ ^cells was not an 'estrogen responsive' gene signature. Rather, it was more likely to represent an underlying stable gene expression pattern characteristic of this differentiated cell population. To investigate this further, we compared lists of estrogen responsive genes reported in studies of estrogen-stimulated normal human mammary epithelial cells [25,26] and breast cancer cell lines with our lists of genes expressed in the epithelial subpopulations [22-24]. The results [see Additional file 18] confirmed that there was little correlation between 'estrogen-responsive' signatures and the genes enriched in the luminal ER^+ ^population. Indeed, many of the gene whose expression was stimulated by estrogen in the breast cancer cell lines were found to be enriched in our basal/myoepithelial population. However, it should be noted that the well-known directly estrogen responsive genes *KRT19 *and *PGR*, were not found to be upregulated in most of the published datasets.

### Identification of basal/myoepithelial cells as key mediators of paracrine signalling

Having identified key processes likely to be occurring within each of the three populations, we next investigated how the populations might be interacting with each other. Mammary epithelial biology is characterised by the conversion of systemic hormone signals into local growth factor signals which stimulate stem cell proliferation and differentiation of daughter cell types. Whilst some of these paracrine interactions have been studied in depth [15,17,41], the broad extent of paracrine networks within the mammary epithelium remains unknown. We therefore queried the gene expression array data for genes potentially involved in paracrine signalling, either as receptors or ligands (where there was a conflict between the gene expression array and qPCR data, the distribution predicted by the qPCR data was favoured). A number of genes were identified which fulfilled these criteria [see Additional file 19]. Remarkably, they showed that the basal/myoepithelial cells have more than double the complement of cell-surface receptors and ligands than either of the two luminal populations. Of particular interest, the basal/myoepithelial cells expressed the genes for the Notch family ligands, *Jag1*, *Jag2 *and *Dll1 *[42] whilst the gene for the Notch family receptor *Notch3 *[43] was expressed in both the luminal populations. Wnt family ligands were expressed by all three populations, although each cell type expressed a different complement of *Wnt *genes and only the basal/myoepithelial cells expressed the genes for the *Frizzled *receptors [44].

It is well established that Egf receptor family members and Egf family ligands are important for mammary gland development and breast cancer [45,46]. In particular, the paracrine role of Amphiregulin (Areg) is well described [15,41,47,48]. Our analysis confirmed that luminal ER^+ ^mammary epithelial cells expressed the *Areg *gene but also showed that the genes for two other family members, Betacellulin (*Btc*) [49] and Epigen (*Epgn*) [50], were expressed in this cell type and *Btc *was also expressed in the luminal ER^- ^cells. Interestingly, only one Egf receptor family member, *Erbb3 *[51], was found by gene expression array and qPCR analysis to be differentially expressed in the normal mammary epithelium. It was present in both the luminal epithelial populations but not in the basal/myoepithelial cells.

This analysis extends previous findings of paracrine signalling within the mammary epithelium and emphasises the complexity of the interacting signalling networks. These include Wnt and Notch signalling, the Egf family, Fgf signalling, other receptor tyrosine kinases, G-protein coupled receptors, ligands for such receptors, integrins and ephrins/Eph receptors all of which are differentially expressed between the cell populations. In particular, the numbers of ligands and receptors expressed by the basal/myoepithelial cells indicates that this population is a key mediator of the paracrine signalling networks within the mammary epithelium.

### Identification of differentially regulated transcription factors within the mammary epithelium

Transcriptional regulators are key cell-intrinsic factors in lineage selection and cell fate decisions in stem – differentiated cell hierarchies [52,53]. Therefore, the gene expression array data set was interrogated to identify differentially expressed factors which may regulate transcription [see Additional file 19]. The expression of a subset of these was analysed by qPCR (Figure 6B). As with the potential paracrine interactions, the basal/myoepithelial cells differentially expressed many more transcriptional regulators (fifty-two) than the other two populations. The ER^- ^luminal cells differentially expressed twenty-one transcription factors but only seven transcription factors were differentially expressed in the luminal ER^+ ^cells. Three transcription factors were found to be expressed in both the basal/myoepithelial and luminal ER^- ^populations, one was common to both the basal/myoepithelial and luminal ER^+ ^cells and two were found in all three populations.

**Figure 7:**
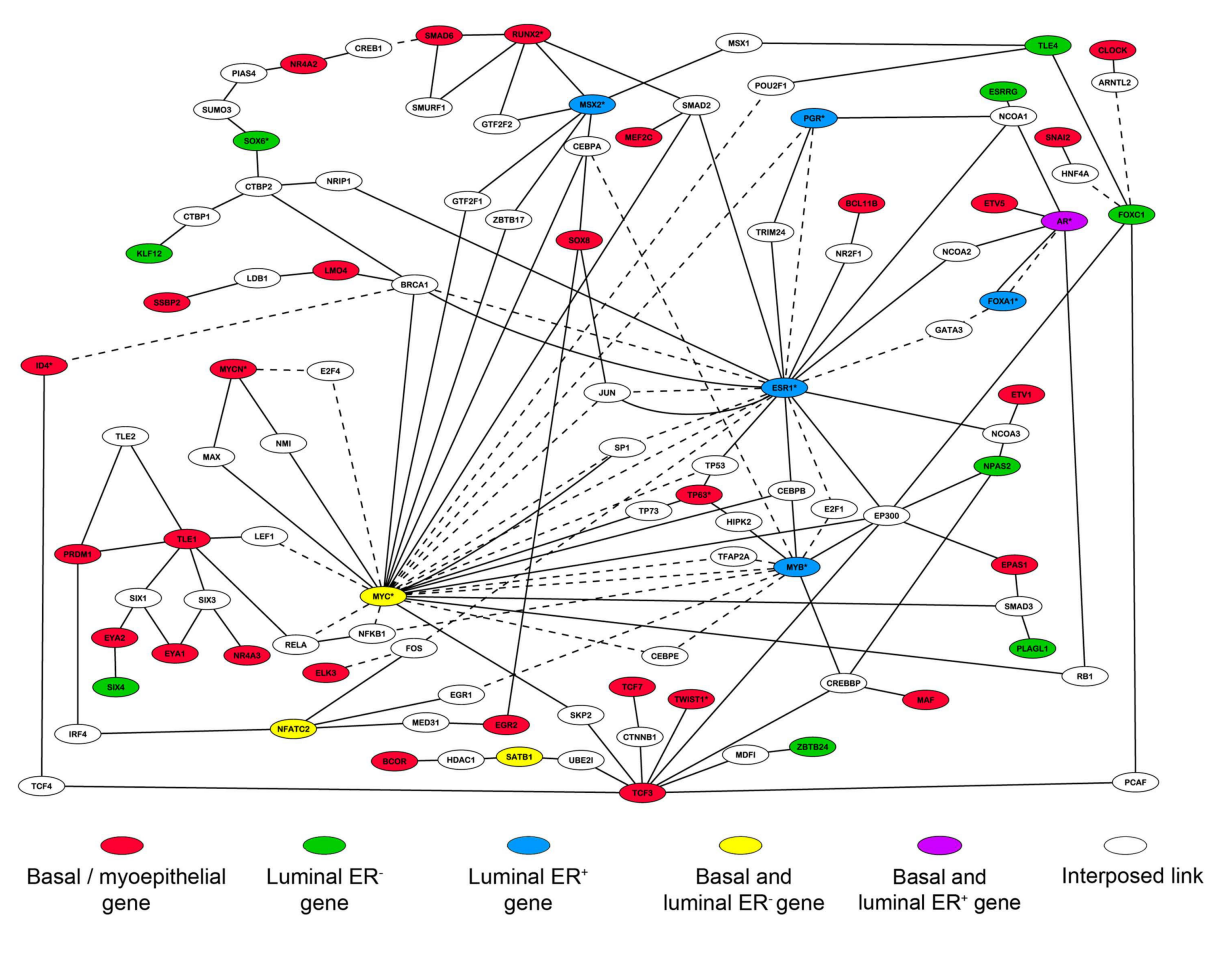
**Interaction mapping of differentially expressed transcriptional regulators suggests *Esr1 *and *Myc *control the balance between basal and luminal differentiation**. Interaction map generated using in-house ROCK database and then manually curated. Genes are colour-coded as indicated. Interaction data based on physical interactions (solid lines) and transcriptional interactions (dashed lines).

In common lineage progenitors, functional mutual repression and auto-stimulation by transcription factors can facilitate bilineage cell fate decisions [54]. Once the cell fate decisions have been executed, continued function of different subsets of the factors active in the progenitors are required to maintain the different differentiated cell lineages in stable fates [53]. Thus, modelling interactions between lineage-specific transcription factors can elucidate cell fate decision processes occurring in progenitor cells. Therefore, to begin to understand how interactions between lineage specific transcription factors may influence cell fate choices in the mammary epithelium, an interaction network was built. Transcription factors identified as lineage-specific but for which no interaction data exists were excluded. The resulting network (Figure 7) was centred around two main hubs, namely *Myc *and *Esr1*, suggesting that they are key regulators of mammary cell fate [55,56]. Clearly, a great deal of information exists on their interactions and this would bias any interaction network around these two key nodes. Nevertheless, the number of (indirect) interactions that *Myc *makes with the basal-specific factors in particular suggests that it is a key factor in regulating mammary cell fate determination. The small number of luminal ER^+ ^population specific factors and the fact that three of them, *Esr1*, *Pgr *and *Foxa1 *[57], are closely connected also supports a key role for the estrogen receptor and its transcriptional network in regulating luminal ER^+ ^cell fate. As described above, however, the luminal ER^+ ^transcriptional profile is not, in general, an estrogen responsive profile. Therefore, the activity of the luminal ER^+ ^specific transcription factors most likely leads to long term stable cell fate changes and not changes which are responsive to short-term fluctuations in estrogen signalling. Mutual repression and auto-stimulation loops may be one way to achieve this long term stability.

### Sox6 is a determinant of luminal cell fate

To demonstrate that the lineage specific transcription factors we identified can indeed influence cell fate and differentiation, we chose to investigate in more detail the function of *Sox6 *[52], the expression of which was only detectable in one population, the luminal ER^- ^cells (Figure 6B). Primary mouse mammary epithelial cells were transduced in three independent experiments with either a control lentivirus carrying GFP only or a virus carrying the *Sox6 *gene plus GFP. The transduced cells were then split between *in vitro *and *in vivo *assays.

**Figure 8:**
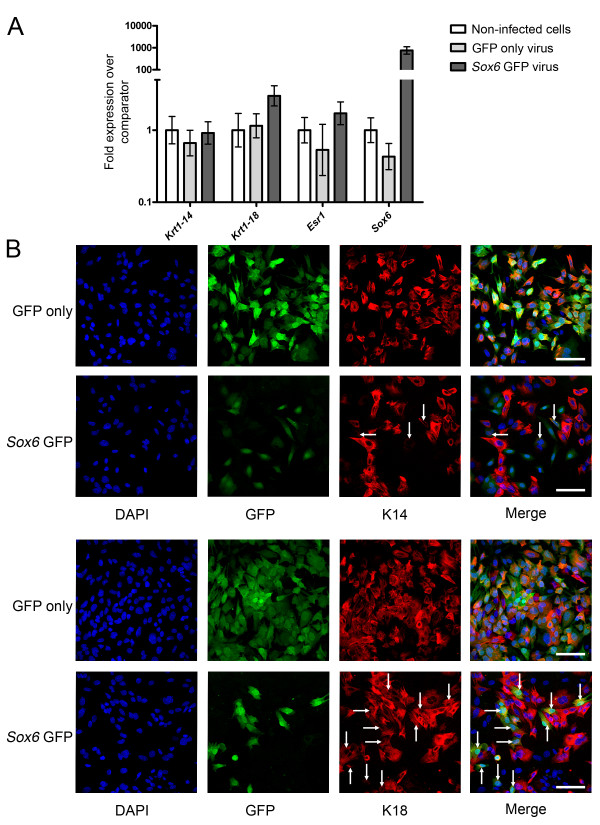
***Sox6 *over-expression maintains luminal differentiation in mammary epithelial cells *in vitro***. A) qPCR analysis of *Krt14*, *Krt18*, *Esr1 *and *Sox6 *expression levels (mean levels from three independent experiments ± 95% confidence intervals) in cells transduced with virus expressing GFP only (light grey bars) or expressing both *Sox6 *and GFP (dark grey bars) compared to non-infected cells (open bars). B) Immunofluorescence staining for keratin 14 (upper two rows) and keratin 18 (lower two rows) expression in primary mouse mammary epithelial cells transduced with lentivirus expressing GFP only or *Sox6 *and GFP. Bars = 30 μm. Arrows indicate keratin positive cells in the *Sox6 *transduced cultures. These are common in the K18 stained cells but rare and only weakly stained in the K14 stained cells [see Additional file 20].

*For in vitro *analysis, cells were cultured for one week and then harvested, GFP^+ ^cells were separated by flow cytometry and RNA isolated from them. The expression of *Sox6*, *Krt14*, *Krt18 *and *Esr1 *was examined by qPCR and compared to expression levels in cultured primary cells which had not been transduced with virus. The data (Figure 8A) demonstrated that *Sox6 *over-expression (approximately 800-fold over-expressed in *Sox6 *GFP virus-transduced cells compared to non-transduced cells) did not significantly alter *Krt14 *gene expression. However, *Krt18 *gene expression was significantly increased in *Sox6 *over-expressing cells compared to non-transduced cells or cells transduced with the control virus (an approximately 3-fold increase in expression levels). *Esr1 *expression levels were also increased in *Sox6 *over-expressing cells, although more modestly (1.7 fold; P < 0.05) [58]. Next, cultured virus-transduced primary cells were stained for keratin 14 (K14) and keratin 18 (K18) expression. When primary mouse mammary cells are isolated and grown in short-term culture, the majority of cells which proliferate are derived from the luminal epithelium. Within 48 hours in culture, these luminal origin cells (which are K14^- ^K18^+ ^*in vivo*) begin to promiscuously express K14 and acquire a K14^+ ^K18^+ ^phenotype [59-61]. It is thought that this represents a de-differentiation event resulting from the cells being removed from their normal environment [62]. Unsurprisingly, therefore, when non-transduced (GFP^-^) and control transduced primary cells were stained for K14 and K18 expression, they were found to be both K14^+ ^and K18^+^. However, *Sox6 *transduced cells were K18^+ ^but showed only weak K14 staining in occasional cells (Figure 8B) [see Additional file 20]. Therefore, *Sox6 *maintained mammary epithelial cells in the luminal epithelial lineage and blocked promiscuous K14 protein expression *in vitro*. However, as *Sox6 *over-expression did not block *Krt14 *gene expression, this cannot be a direct effect on *Krt14 *transcription.

**Figure 9:**
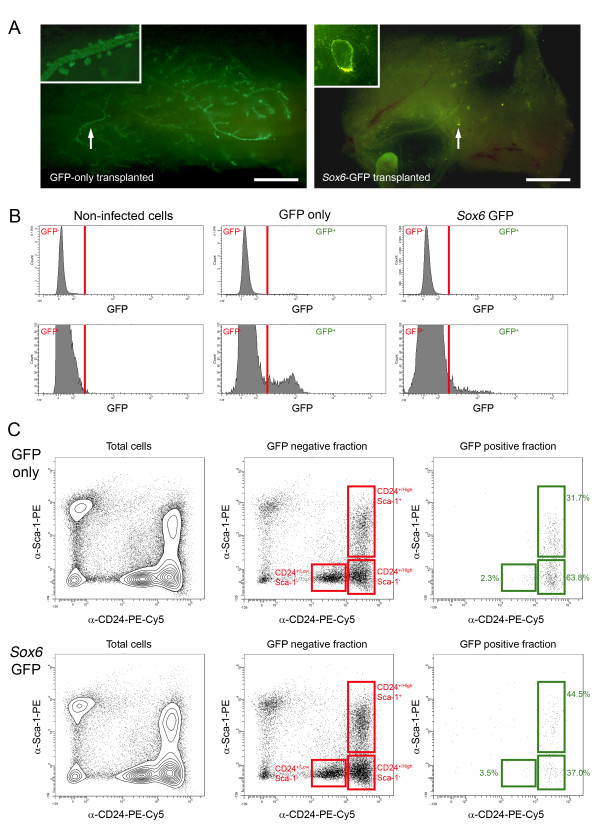
***Sox6 *over-expression maintains luminal differentiation in mammary epithelial cells *in vivo***. A) Wholemounts of fat pads transplanted with mammary epithelial cells transduced with control virus (left) or Sox6 GFP virus (right). Arrows indicate regions magnified in insets (GFP^+ ^duct and alveolar buds in control gland; cyst-like structure in *Sox6 *gland). Bar = 225 μm. B) Flow cytometric analysis of GFP expression in non-infected, wild type cells (left), cells prepared from control transplanted fat pads (centre) and cells prepared from *Sox6 *transplanted fat pads (right). The lower set of histograms are magnifications of the corresponding upper histograms to show the GFP^+ ^cells with greater clarity. C) Anti-CD24 and anti-Sca-1 staining pattern of cells prepared from control transplanted fat pads (upper plots) and *Sox6 *transplanted fat pads (lower plots). The plots for the total cells isolated from the transplanted fat pads and the GFP^- ^and GFP^+ ^fractions of cells isolated are shown. The CD24^+/Low l^Sca-1^-^, CD24^+/High ^Sca-1^- ^and CD24^+/High ^Sca-1^+ ^regions are indicated as are the percentages of GFP^+ ^cells in these regions.

For *in vivo *analysis, cells transduced with either GFP-only or *Sox6*-GFP virus were transplanted into cleared mouse mammary fat pads. After eight weeks, the transplanted fat pads were examined (Figure 9A). In nine out of eighteen fat pads transplanted in three independent experiments with cells transduced with the GFP-only virus, extensive mammary epithelial outgrowths were seen in which both ducts and alveolar buds were GFP labelled. However, no outgrowths were observed in twenty fat pads transplanted in three independent experiments with cells carrying the *Sox6*-GFP, although in two cases, cyst-like GFP-labelled structures were observed.

It was not possible to determine at the wholemount level whether fat pads which did not have extensive GFP^+ ^outgrowths contained scattered GFP-labelled cells incorporated into non-GFP epithelium. Therefore, transplanted fat pads were processed to single cells, labelled with anti-Sca-1 and anti-CD24 antibodies and analysed by flow cytometry to identify GFP^+ ^cells and determine which of the mammary epithelial cell populations they segregated with (Figure 9B, C). Transplanted fat pads from two experiments were pooled for this analysis whilst fat pads from the third transplant experiment were analysed independently. As expected, GFP^+ ^cells could be detected in the preparations derived from these control fat pads (Figure 9B). These control GFP^+ ^cells mainly segregated with the CD24^+/High ^Sca-1^- ^luminal ER^- ^population, although they could also be found in the CD24^+/Low ^Sca-1^- ^basal/myoepithelial and CD24^+/High ^Sca-1^+ ^luminal ER^+ ^cells (Figure 9C). However, GFP^+ ^cells could also be detected in preparations of cells from *Sox6 *GFP virus transplants (Figure 9B). Although only low numbers of GFP^+ ^cells were present, their distribution was shifted towards the CD24^+/High ^Sca-1^+ ^luminal ER^+ ^population (Figure 9C).

## Discussion

We have described a comprehensive transcriptome analysis of three distinct mammary epithelial cell populations, basal/myoepithelial cells, estrogen receptor negative luminal epithelial cells and estrogen receptor positive luminal epithelial cells [5,8]. These data provide new support for the distinct identities of these three populations and, in particular, justify distinguishing between two major subpopulations within the luminal epithelium. We have termed these luminal ER^- ^and luminal ER^+ ^cells as it is expression of the estrogen receptor which makes them most easily distinguishable in tissue sections (Figure 1). However, each of them expresses, in addition, a large number of unique genes which must be of relevance to their *in vivo *functions. It is also clear that there are genes in common to the two luminal populations but which are not expressed by the myoepithelial cells. Expression of these genes, for cytoskeletal proteins (e.g. keratin 18) or tight junction components (e.g. claudins), for instance, must reflect common aspects of luminal epithelial cell function. Most obviously, they are for proteins important in maintaining the structure of the luminal cell layer and the integrity of the lumen itself.

Comparison of our data set with other published data sets for separated myoepithelial and luminal cells [6,26,27,29] showed good concordance between genes previously identified as enriched in mouse mammary myoepithelial and luminal cells [6]. However, there was less agreement with genes previously identified as enriched in human myoepithelial and luminal cells. A number of factors are likely to contribute to these differences. Clearly, species differences could be important. For instance, it is known that while K14 is a basal/myoepithelial cell marker and K18 a luminal epithelial cell marker throughout the mouse mammary epithelium and in the ducts of the human breast, in the Terminal Ductal Lobuloalveolar Units of the human breast, K14 can be expressed by the luminal epithelial cells [63]. Furthermore, technical differences could influence the outcome of the analyses. In particular, it should be noted that Stingl and colleagues used the same Affymetrix platform as ourselves for their mouse analysis [6]. Finally, comparing three distinct populations against each other, rather than just two, improves the contrasts between the populations and enables more population specific genes to be detected. For example, a gene which is present in the basal/myoepithelial cells and one of the luminal populations, but not the other, may not be detected as being significantly differentially expressed when only myoepithelial and total luminal cells are compared. However, when all three populations are compared against each other, the contrast with the luminal population from which the gene is absent enables the differential expression of the gene to be detected.

### A novel functional cell type in the mammary epithelium

The nature of the luminal ER^- ^population as a discrete entity has been confirmed by its uniform staining profile with the 33A10 antibody [8]. However, this population appeared to contain within it further distinct functional cell types. Use of network interaction mapping on the transcriptomic profiles of the luminal ER^- ^cells identified a Toll-like receptor (TLR) signalling module including genes for the three components of the bacterial lipopolysaccharide (LPS) receptor, *Tlr4*, *CD14 *and *Ly96*, as well as downstream transducers of Toll-like receptor signalling such as *Irak2 *and the pro-inflammatory cytokine *Tnf*, which is a TLR signalling target [38] (Figure 4 and 5). Flow cytometric analysis of the mammary epithelium using anti-CD14 and anti-CD61 (a mammary progenitor cell marker) [11] identified a small number of CD61^+ ^cells in the CD24^+/Low ^Sca-1^- ^basal population and four distinct subpopulations within the luminal ER^- ^cells, namely CD61^+^, CD61^+ ^CD14^+^, CD14^+ ^and double negative cells. This raises the possibility of a differentiation hierarchy in which basal stem cells generate basal CD61^+ ^progenitors which then become CD61^+ ^progenitors with luminal characteristics. These then either lose CD61^+ ^and become the double negative cells (although presumably expressing other markers as yet undefined) or first acquire CD14 expression, becoming CD61^+ ^CD14^+ ^progenitors, and then lose CD61 expression to become the CD14^+ ^population. If this hypothesis is correct, it suggests that the luminal ER^- ^population contains two distinct functional cell types, the double negative cells and the CD14^+ ^cells.

The function of the double negative cells remains unclear. However, given that the expression of all components of the LPS receptor can be found in the luminal ER^- ^population, it is likely that the CD14^+ ^cells have a distinct function within the mammary epithelium as non-professional immune cells. Note that CD45^+ ^cells were excluded from this analysis, so these are unlikely to be contaminating haematopoietic cells. Milk is an excellent growth medium for bacteria and it would be evolutionarily advantageous to have a cell type present in the breast capable of indicating the presence of bacterial contamination through Toll-like receptor signalling pathways. However, it is also likely that over-stimulation of this pathway in CD14^+ ^mammary epithelial cells would lead to serious inflammation and would therefore be the cause of mastitis.

The presence of the distinct subpopulations within the luminal ER^- ^cells indicates that the gene expression profile of this population is derived from a mixture of different cell types. However, as the luminal ER^- ^cells do all share at least one distinctive marker (high levels of expression of the epitope bound by the 33A10 antibody) [8] it is likely that the luminal ER^- ^profile includes genes whose expression is common to all the cells of this subpopulation, as well as genes expressed in subsets of its cells. The basal/myoepithelial cell population is also a mixed population. However, > 90% of these cells express both keratin 14 and α-isoform smooth muscle actin and are thus differentiated myoepithelial cells [5]. Therefore, the gene expression profile of this population is largely a myoepithelial cell profile. The luminal ER^+ ^population is also unlikely to represent a completely uniform cell population but as the majority of the cells are strongly keratin 18 positive and express the estrogen receptor [5], the gene expression profile of this population will be dominated by this single cell type.

### Myoepithelial cells are key regulators of paracrine signalling

Transcriptome and network interaction analysis of the basal/myoepithelial cells identified key processes of these cells as cytoskeletal function and extracellular matrix production and interactions. Given what is known about the contractile function of these cells in lactation and their position in the mammary gland between the luminal epithelium and the basement membrane around the ducts and lobulo-alveolar structures, these results were reassuring. However, what was remarkable was that the genes characteristic of this population included more than double number of genes for proteins involved in cell – cell signalling than were characteristic of the two luminal populations combined. This suggests a key role for myoepithelial cells in mediating paracrine and juxtacrine cell – cell interactions in the mammary epithelium. Of particular interest were the expression patterns of Wnt and Notch signalling pathway components, known to be important in mammary gland development [64,65], which suggested a directionality of Notch signalling from basal to luminal and a directionality of Wnt signalling from luminal to basal. Notably, Notch signalling was recently shown to be important for determining luminal cell fate [19], possibly through regulation of luminal progenitors [18]. Activated Wnt signalling has also been shown to increase the stem/progenitor fraction of basal mammary epithelial cells in MMTV-Wnt1 transgenic mice [3].

Another gene from a family important for mammary development that, like *Notch3*, was expressed in both the luminal populations but not the basal/myoepithelial cells was *Erbb3*. A member of the epidermal growth factor receptor family, Erbb3 most effectively binds the neuregulins Nrg1 and Nrg2, but it has no intrinsic signalling activity of its own. It must therefore operate as a heterodimer with other family members. However, signalling complexes containing *Erbb3 *have a strong propensity to activate the phosphoinositide-3-kinase (PI3K) signalling pathway due to the presence of six binding sites for the p85 SH3 adaptor subunit of PI3K [51]. *Erbb3 *knockout animals were embryonic lethal but reduction of *Erbb3 *expression in the mammary epithelium caused a reduction in terminal end bud numbers, branching and ductal density [51,66]. Previous reports of *Erbb3 *expression have been inconsistent [67,68], most likely due to variable antibody quality, however, the ductal outgrowth defects that occur when *Erbb3 *expression is reduced, together with the observation that implantation of Nrg1-soaked pellets induced ductal elongation at puberty [69], support a role for Erbb3 in pubertal mammary development. Whether or not Erbb3 activation has the same consequences in both the luminal ER^+ ^and luminal ER^- ^cells remains to be determined and may depend on differential expression of dimerisation partners not detected by the microarray assays.

### Mammary epithelial cell subpopulations have distinct transcription factor profiles

Mutual interactions between transcription factors associated with different cell lineages and involving positive and negative feedback loops have been demonstrated to be able to maintain haematopoietic cells in a small number of particular cell fates ('stable states') when an apparently large number of potential intermediate fates are available [54]. Transcription factors in the mammary epithelium which have the potential to interact but which are apparently expressed in different populations are therefore of particular interest in the regulation of mammary epithelial cell fate. We built a gene network to predict such interactions and identified a number of these which are potentially important. The most obvious is between *Esr1 *(luminal ER^+^) [56], *Trp63 *(basal) [70] and *Myc *(basal and luminal ER^-^) [55] and given the large number of transcription targets they share, it is likely that these three are key factors in determining cell fate in the mammary gland. However, there are also other interacting factors of interest such as the *Runx2 *(basal) [71] – *Msx2 *(luminal ER^+^) [72] pair, the *Eya2 *(basal) – *Six4 *(luminal ER^-^) pair [73] and the *Foxa1 *(luminal ER^+^) [57] – *AR *(basal and luminal ER^+^) [74] – *Etv5 *(basal) [75] triplet. Modelling these interactions in order to make predictions about how transcription factor behaviour determines mammary cell fate is an important challenge for the future.

### Sox6 is a determinant of luminal cell fate in the mammary gland

In order to model these interactions, functional data on each individual factor will be required. Given the large number of factors of interest, a relatively rapid throughput assay will be required, ruling out the use of transgenic or knockout mice. Therefore, to demonstrate that functional information on the role in determining mammary cell fate of the transcriptional regulators identified in this study can be relatively rapidly generated, and to provide at the same time the first data required for this modelling, we examined the function of Sox6. A member of the SoxD group of the Sry-related, high mobility group box transcription factor family, Sox6 has two dimerisation domains and the HMG box domain, but no transactivation or transrepression domains [52]. Its action as an activator or repressor of transcription depends, therefore, on its binding partners [52] and it has been shown both to repress specification and differentiation of oligodendrocytes during gliagenesis [76] and promote differentiation and maturation of chondrocytes during skeletogenesis [52,77]. *Sox6 *has been shown to be upregulated in the mammary gland by 2-methoxyestradiol treatment [78] but its role in mammary differentiation has not been previously investigated.

In this, study, *Sox6 *was specifically expressed in the luminal ER^- ^cells and was undetectable in the basal and luminal ER^+ ^cells. Over-expression of *Sox6 in vitro *caused an increase in *Krt18 *luminal marker gene expression and a slight, but significant increase in *Esr1 *expression. It did not change *Krt14 *gene expression. However, staining of the cells with antibodies to either K14 or K18 showed that while control primary mammary cells in short-term culture promiscuously expressed both K14 and K18 (as previously described) [59-61], *Sox6 *over-expressing cells were K18 positive but K14 negative. Therefore, *Sox6 *over-expression maintained the mammary epithelial cells in the luminal phenotype and prevented promiscuous K14 protein expression. Cleared fat pad transplant of *Sox6 *over-expressing primary cells mixed with wild-type cells failed to generate extensive GFP^+ ^outgrowths, suggesting that *Sox6 *over-expression may block transplantation activity. However, rare GFP^+ ^cells could be detected in cells isolated from *Sox6 *transplanted fat pads and analysed by flow cytometry. The phenotype of these rare cells was biased toward the luminal ER^+ ^population. It is unlikely that this was due to transduction of different cell populations by the control and *Sox6 *viruses, as viral tropism is determined by envelope proteins and these are coded by the viral packaging plasmids, which were identical for the two viruses. The small numbers of cells which could be detected and the caveats associated with over-expression studies mean that caution must be exercised in interpreting these data. However, they are consistent with the *in vitro *data that *Sox6 *is involved in promoting or maintaining a differentiated luminal phenotype, a corollary of which is that it blocks stem cell behaviour (transplantation). A more detailed understanding of the function and mechanism of Sox6 action in the mammary epithelium must await knockdown and inducible over-expression studies. Nevertheless, our data are the first to suggest that Sox6 has a role in cell fate determination in the mammary epithelium.

## Conclusion

This transcriptome analysis of mammary epithelial cell subpopulations has provided a framework for future studies of normal mammary epithelial cell homeostasis and the molecular pathology of breast disease. First, it has confirmed the existence of distinct luminal epithelial cell lineages with distinct gene expression patterns. Second, it has identified a novel functional specialisation within the mammary epithelium, that of non-professional immune cell. Third, it has highlighted the complexity of the potential paracrine interactions occurring within the mammary gland. Fourth, it has identified cell-type specific transcriptional regulators with potential roles in mammary epithelial cell lineage specification and fate determination and has shown how these factors are likely to operate in a complex network. Last, it has shown that one of the factors identified, *Sox6*, may be a determinant of luminal cell fate in the mammary epithelium. Future studies will use these data to explore the contribution of the three epithelial cell types to different tumour phenotypes. They will also focus on the role of the transcription factor network in cell fate choice and cellular homeostasis to model how perturbations in this network may lead to cancer.

## Methods

### Preparation and flow cytometry of single mammary cell suspensions

All animal work was carried out under UK Home Office project and personal licences following local ethical approval and in accordance with local and national guidelines. Fourth mammary fat pads were harvested from 10 week old virgin FVB mice. Single mammary cells suspensions were prepared as described [4,5]. Mammary cell suspensions at 10^6 ^cells/ml were stained with anti-CD24-FITC (clone M1/69, BD Biosciences, Oxford, UK, 0.5 μg/ml), anti-CD45-PE-Cy5 (clone 30-F11, BD Biosciences, 0.25 μg/ml) and anti-Sca-1-PE (clone D7, BD Biosciences, 0.5 μg/ml) as described [4,5]. For anti-CD14 and CD61 staining, cells were stained with anti-CD14-PE (clone rmC5-3, BD Biosciences, 1.0 μg/ml), anti-CD61-FITC (clone 2C9.G2, BD Biosciences, 2.5 μg/ml), anti-CD24-PE-Cy5 (clone M1/69, eBioscience, Insight Biotechnology Limited, London, UK, 0.6 μg/ml), anti-Sca-1-APC (clone D7, eBioscience, 1.0 μg/ml) and anti-CD45-PE-Cy7 (clone 30-F11, BD Biosciences, 1.0 μg/ml). For analysis of fat pads transplanted with lentivirus-transduced cells, anti-CD24-PE-Cy5, anti-Sca-1-PE and anti-CD45-PE-Cy7 were used. Cells were sorted at low pressure (20 psi using a 100 μm nozzle) on a FACSAria (Becton Dickenson, Oxford, UK) equipped with violet (404 nm), blue (488 nm), green (532 nm), yellow (561 nm) and red (635 nm) lasers. Both cell sample and collection tubes were maintained at 4°C. Single stained samples were used as compensation controls. Dead cells, CD45^+ ^leukocytes and non-single cells were excluded as described [4,5].

### cDNA microarray gene expression analysis on freshly isolated mammary epithelial cells

Freshly sorted mammary epithelial subpopulations were resuspended in RLT buffer (Qiagen, Crawley, West Sussex, UK) and stored at -80°C until required for RNA extraction. Total RNA was extracted using a RNeasy MinElute Kit (Qiagen), according to the manufacturers' instructions from CD24^+/Low ^Sca-1^-^, CD24^+/High ^Sca-1^- ^and CD24^+/High ^Sca-1^+ ^cells isolated from three independent preparations of virgin mammary tissue. RNA quantity and purity was tested in an Agilent 2100 Bioanalyser (Agilent, Wokingham, Berkshire, UK). RNA was converted to cDNA using an oligo d(T) primer, amplified and biotin labeled using the Ambion MessageAmp II Biotin Enhanced kit (Applied Biosystems, Warrington, Cheshire, UK). The samples were fragmented to 35–200 bp and hybridized to Affymetrix Mouse Expression MOE430 2.0 arrays  for 16 hours at 45°C. The arrays were washed, labeled using an antibody bound to phycoerythrin and scanned according to the manufacturer's protocols. Primary array data, SAM outputs and normalised data complying with MIAME standards are available through ROCK [79].

### Bioinformatic analysis

Expression data were normalised and summarised by robust multi-array analysis (RMA) using the Affymetrix package in the statistical environment R 2.5 [80]. Probe sets with a standard deviation > 0.25 were used for a multiclass Significance Analysis of Microarray (SAM; version 3 Excel add-in) [30] to determine if their mean expression was different across the three subpopulations. Genes determined by this analysis to have a relative abundance of 2 or more in a population were considered characteristic of that population. Clustering analysis was carried out using CLADIST [81].

Data mining for genes of interest in paracrine signalling interactions or as transcription factors was carried out by uploading the lists of genes differentially expressed in the cell subpopulations into the DAVID Bioinformatics Resource [82] and searching the SP_PIR_KEYWORDS and GOTERM_BP_ALL lists.

Network interactions maps were provided by uploading gene lists into a web-based in-house bioinformatics package and database, ROCK, developed based on the pSTIING server [81]. Interaction maps generated were manually curated to ensure no interpolated connecting genes were inappropriately added (so that, for instance, *ESR1 *did not appear as a connecting gene in the basal/myoepithelial network map).

### Quantitative PCR analysis

For quanitative PCR-based gene expression analysis, cDNA synthesis was carried out using a Sensiscript RT kit (Qiagen). Up to 50 ng of RNA was transcribed into cDNA using an oligo dT^n ^primer (Promega, Southampton, Hampshire, UK) per reaction. 0.5 μl of cDNA was used per qPCR reaction. Each analysis reaction was performed in triplicate on fresh RNA samples collected separately to those used for the microarray analysis. *β-Actin *was used as an endogenous control throughout all experimental analyses. Gene expression analysis was performed using TaqMan Gene Expression Assays (Applied Biosystems, Warrington, Chesire, UK) on an ABI Prism 7900HT sequence detection system (Applied Biosystems) [see Additional file 21]. Analysis was performed using the ^Δ-Δ^Ct method to determine fold changes ± 95% confidence limits in gene expression across three independently isolated samples relative to a comparator in a 'round robin' method in which each population was used in turn as the comparator. With this method, the data was separately plotted for each of the two non-comparator populations against the comparator. The non-comparator population was used to order the dataset on each graph in descending levels of relative expression from left to right. The point after which the differences in expression level between the two populations ceased to be significant (when the confidence intervals of one population overlap the mean of the other) [58] was plotted (vertical dotted line). All comparator population genes which fall to the right hand side of the vertical line in both graphs have similar or elevated levels of expression in the comparator population compared to both the non-comparator populations. Such genes were considered to be characteristic of the comparator population. Note that in cases where expression of the gene being analysed could not be detected in the comparator sample, an artificial Ct value of 40 was assigned purely to make the comparisons. The gene was still recorded as undetectable in the presented data.

### Lentivirus production

*Sox6 *cDNA was kindly provided by Veronique Lefebvre (Cleveland Clinic Lerner Research Institute, Cleveland, Ohio) in pcDNA3.1 and was subcloned into pWPI lentivirus expression vector (Tronolab) [83] by *PmeI *digest. Viral supernatants were generated by co-transfection of the expression vector and two packaging vectors (psPAX2 and pMD2.G) into HEK293T cells. Cells were refed with fresh medium (Dulbecco's Modified Eagle's Medium, DMEM; Invitrogen, Paisley, UK) plus 10% foetal calf serum (FCS; PAA Laboratories, Yeovil, Somerset, UK) after 24 hours. Supernatants were harvested 48 and 72 hours after transfection and checked for absence of replication-competent virus. Supernatants were stored at -80°C until use.

### Mammary epithelial cell transduction and transplantation

Freshly isolated primary mouse mammary epithelial cells were resuspended at 1 × 10^6 ^cells/ml in viral supernatant and plated at 1 ml/well in ultra-low attachment 24-well plates (Corning, Fisher Scientific, Loughborough, Leicestershire, UK) [84]. After 16 hours, the cells (now in clumps) were washed and replated in 1:1 DMEM: Ham's F12 medium (Invitrogen) with 10% foetal calf serum, 10 ug/ml insulin (Sigma), 100 ng/ml epidermal growth factor (Sigma) and 10 ng/ml cholera toxin (Sigma) (growth medium) [60] and transferred to ultra-low attachment 6-well plates (Corning). After a further 24 hours, the majority of the cell clumps were injected into cleared fat pads of 3-week old syngeneic FVB mice as described [4]. A proportion, however, were transferred to glass coverslips and/or normal tissue culture plastic and maintained in growth medium under low oxygen culture conditions [59,60] for one week. After this time, cells on plastic were trypsinized and flow sorted to isolate GFP^+ ^cells for qPCR analysis. Cells on coverslips were fixed in 4% paraformaldehyde in PBS, stained for either keratin 14 (clone LLOO2; Abcam, Cambridge, UK) or keratin 18 (clone Ks18.04; Progen, Heidelberg, Germany), expression by standard techniques [59], counterstained with DAPI and then examined on a TCS SP2 confocal microscope with an Acousto-Optical Beam Splitter and lasers exciting at 405, 488, 555 and 633 nm (Leica Microsystems, Milton Keynes, UK).

### Analysis of transplanted fat pads

Transplanted fat pads were analysed after eight weeks. Fat pads were stretched on glass slides and then examined under epifluorescent illumination and photographed. Fat pads injected with control transduced cells or cells transduced with *Sox6 *virus were then processed as separate batches to single cells as described [4,5], stained for CD45, CD24 and Sca-1 expression and analysed by flow cytometry.

### Multiple immunofluorescence staining of mouse mammary gland sections

The protocol for multicolour immunostaining of paraffin embedded tissue has been recently described [85]. In brief, mammary fat pads from ten-week old virgin female FVB mice were fixed in 4% buffered formalin, overnight. Following standard processing, antigen retrieval was carried out on 4 μm paraffin-embedded sections by boiling in 0.01 M citrate buffer, pH 6, for 18 minutes in a microwave (900 W). Sections were then blocked for 1 hour in MOM mouse Ig blocking reagent (Vector Laboratories, Peterborough, UK; 9 μl stock MOM Ig blocking reagent in 250 μl TBS) and 30 minutes in DAKO protein block (DAKO, Ely, Cambridgeshire, UK). Sections were stained with antibodies against K14 (0.26 μg/ml; mouse IgG3 clone LLOO2; Abcam, Cambridge, UK) and K18 (diluted 1:2 from ready-to-use solution; mouse IgG1 clone Ks18.04; Progen, Heidelberg, Germany) or keratin 14 and ER (mouse IgG1 clone 1D5; 1:40 dilution; DAKO), overnight at 4°C. They were then stained with isotype-specific goat anti-mouse secondary antibodies conjugated to Alexa Fluor 488 or 555 fluorochromes, counterstained with DAPI and mounted in Vectashield (H1000; Vector Laboratories) mounting medium. Sections were examined at room temperature on the TCS SP2 confocal microscope. Multicolour images were collected sequentially in three channels. Images were captured using the Leica system and Leica TCS image acquisition software. Co-localisation overlays were generated using TCS software. Control single stained sections in which either the primary antibody was left out or the primary antibody was combined with the wrong secondary antibody showed no staining.

## Abbreviations

APC: Allphycocyanin; DAPI: 4,6-diamidino-2-phenylindole dihydrochloride; DMEM: Dulbecco's Modified Eagle's Medium; EMT: Epithelial-mesenchymal transition; ER: Estrogen Receptor; FCS: Foetal Calf Serum; FITC: Fluorescein Isothiocyanate; K14: Keratin 14; K18: Keratin 18; L15: Leibowitz L15 medium; LPS: Lipopolysaccharide; PE: Phycoerythrin; PE-Cy5: Phycoerythrin-Cy5; PE-Cy7: Phycoerythrin-Cy7; qPCR: Quantitative real time rtPCR; TLR: Toll-Like Receptor.

## Authors' contributions

HK collected primary mammary cell samples, isolated RNA, carried out qPCR analyses, helped analyse microarray data, carried out fat pad transplants and analysed transplant results. JR prepared virus and assisted with fat pad transplants. FM developed immunostaining protocols and stained and analysed tissue sections. AG carried out SAM analysis on array data. MZ and CM carried out clustering and network analysis on array data. MS designed the study, collected primary mammary cell samples, analysed microarray data, stained primary mouse cells *in vitro *and wrote the manuscript.

## Supplementary Material

Additional file 1 **qPCR analysis of *Krt14*, *Krt18 *and *Esr1 *expression in mammary epithelial subpopulations**. The data describes qPCR analysis of expression of *Krt14*, *Krt18 *and *Esr1 *in triplicate independent samples of CD24^+/Low ^Sca-1^- ^cells, CD24^+/High ^Sca-1^- ^cells and CD24^+/High ^Sca-1^+ ^cells. Each data point is the mean level of expression, ± 95% confidence intervals, across the three samples of that population relative to the comparator sample. A 'round robin' comparison was used as described in the Methods. Genes considered to be characteristic of the comparator population are indicated next to each pair of graphs. *Krt14 *expression was undetectable in the CD24^+/High ^Sca-1^- ^and CD24^+/High ^Sca-1^+ ^cells. *Krt18 *expression was undetectable in the CD24^+/Low ^Sca-1^- ^cells. Click here for file

Additional file 2 **Relative expression levels of 2182 Affymetrix probes across three virgin mouse mammary epithelial subpopulations**. The spreadsheet gives the relative expression levels for all differentially expressed probes across all three populations. Expression levels are indicated by a relative abundance score for each populations. A high positive value indicates expression at a high level, a low negative score indicates very low expression levels. The Affymetrix probe ID, Gene Symbol and q-value (indicating the % false discovery rate) are also indicated. Click here for file

Additional file 3 **Full heat map and hierarchical cluster analysis of differential gene expression across virgin mammary epithelial populations**. The image shows heat map clustering of differentially expressed genes across the three cell populations. Red indicates high expression, green indicates low expression. Click here for file

Additional file 4 **Genes characteristic of basal/myoepithelial cells**. The table shows all 861 genes in the basal/myoepithelial population with an abundance score of 2 or more when the 1427 differentially expressed gene set was sorted by descending abundance scores in the basal/myoepithelial population. Such genes were considered characteristic of the population. Where differential gene expression was indicated by more than one probe, an average value for each of the contrasts across the probes was calculated. The number of probes is indicated. Click here for file

Additional file 5 **Genes characteristic of luminal ER^- ^cells**. The table shows all 326 genes in the luminal ER^- ^population with an abundance score of 2 or more when the 1427 differentially expressed gene set was sorted by descending abundance scores in the luminal ER^- ^population. Such genes were considered characteristic of the population. Where differential gene expression was indicated by more than one probe, an average value for each of the contrasts across the probes was calculated. The number of probes is indicated. Click here for file

Additional file 6 **Genes characteristic of luminal ER^+ ^cells**. The table shows all 488 genes in the luminal ER^+ ^population with an abundance score of 2 or more when the 1427 differentially expressed gene set was sorted by descending abundance scores in the luminal ER^+ ^population. Such genes were considered characteristic of the population. Where differential gene expression was indicated by more than one probe, an average value for each of the contrasts across the probes was calculated. The number of probes is indicated. Click here for file

Additional file 7 **Summarised gene expression microarray and qPCR gene expression analysis for 58 test genes**. The table shows a comparison between the gene expression patterns determined by microarray analysis and those determined by qPCR. The gene expression microarray relative abundance scores are summarised as follows: --- = -32 to -22, -- = -22 to -12, - = -12 to -2, +/- = -2 to +2, + = 2 to 12, ++ = 12 to 22, +++ = 22 to 32, ++++ = 32 to 42. Where more than one identifier for a gene scored as differentially expressed, the mean score across all the identifiers was used to determine the summarised microarray score. The summarised score was in turn used to define the array-based expression pattern, with a score of +, ++, +++ or ++++ indicating that a gene was expressed in a particular population. In some cases, the genes were expressed in more than one population. The summarised qPCR expression pattern is based upon the patterns of gene expression determined from Figure 3. Whether or not the array and qPCR based analyses show concordance in their assignment of expression patterns is indicated. NDE = no differential expression in microarray. N/A = comparison cannot be made as qPCR probe failed. *In these comparisons, technical failure of the Affymetrix probe cannot ruled out. Click here for file

Additional file 8 **Comparison of numbers of genes identified in common between basal/myoepithelial, luminal ER^- ^and luminal ER^+ ^cells and previously published datasets**. The table compares previously published datasets with the subpopulation specific genes identified in the current analysis. Published lists of genes [27,28], probes [6] or SAGE tags [29] significantly differentially expressed between mouse basal mammary stem cell enriched/myoepithelial cells compared to *in vitro *colony forming cells (luminal cells) [6], human CD10^- ^CD44^+ ^basal cells compared to CD24^+ ^luminal cells [29] or human CD10^+ ^myoepithelial cells compared to EMA^+ ^luminal cells [27,28] were condensed to remove multiple probes or tags against the same gene and to identify only well-annotated genes. The distribution of the differentially expressed genes in the basal/myoepithelial, luminal ER^- ^and luminal ER^+ ^gene lists from the current study was then determined. Click here for file

Additional file 9 **List of genes common to basal/myoepithelial cells and previously published basal or myoepithelial datasets**. The table lists those basal/myoepithelial genes found in previously published datasets which were also found the basal/myoepithelial population in the current study. Click here for file

Additional file 10 **List of genes common to luminal cell subpopulations and previously published luminal datasets**. The table lists those luminal genes found in previously published datasets which were also found the luminal populations in the current study. Click here for file

Additional file 11 **Network interaction map for basal/myoepithelial specific genes**. Interaction data between basal/myoepithelial specific genes based on physical interactions (black lines) and interactions in complexes (brown lines) with no interpolated genes used. The nodes are colour coded to indicate relative strengths of expression of the gene within the cell population. Brighter reds indicate highest levels of expression. Darker reds indicate genes less strongly expressed (although still with enriched expression within the population compared to the other cell types). Click here for file

Additional file 12 **Network interaction map for luminal ER^- ^specific genes**. Interaction data for luminal ER^- ^specific genes based on physical interactions (solid lines) and transcriptional interactions (dashed lines). The nodes are colour coded to indicate relative strengths of expression of the gene within the cell population. Brighter reds indicate highest levels of expression. Darker reds indicate genes less strongly expressed (although still with enriched expression within the population compared to the other cell types). White nodes indicate interpolated genes used by the network mapping software to extend and link the network. Click here for file

Additional file 13 **Network interaction map for luminal ER^+ ^specific genes**. Interaction data for luminal ER^+ ^specific genes based on physical interactions (solid lines) and transcriptional interactions (dashed lines). The nodes are colour coded to indicate relative strengths of expression of the gene within the cell population. Brighter reds indicate highest levels of expression. Darker reds indicate genes less strongly expressed (although still with enriched expression within the population compared to the other cell types). White nodes indicate interpolated genes used by the network mapping software to extend and link the network. Click here for file

Additional file 14 **Identification of prominent modules of differentially expressed genes in the luminal ER^- ^and luminal ER^+ ^networks**. The results of the first, second and third pass analyses for network modules in the luminal ER^- ^and luminal ER^+ ^networks are shown. Rectangular nodes are first pass nodes, octagonal nodes are second pass nodes and small green oval nodes are third pass nodes. Thick red lines are first pass connections, medium size red lines are second pass connections and thin red lines are third pass connections. Black rectangles indicate differentially expressed hubs for which no modules could be built. Coloured rectangles indicate module groupings of differentially expressed genes. Solid lines indicate physical interactions, dotted lines transcriptional interactions. Click here for file

Additional file 15 **Topology of interaction modules within the luminal ER^- ^network**. Luminal ER^- ^interaction modules are shown projected on to the luminal ER^- ^network. First, second and third pass nodes are indicated as coloured rectangular, octagonal and oval nodes respectively. First, second and third pass connections are indicated as thick, medium and thin red lines respectively. Solid lines indicate physical interactions, dotted lines transcriptional interactions. The different colourings of the first and second pass nodes indicate module groupings of differentially expressed genes. Third pass nodes are coloured green. Click here for file

Additional file 16 **Topology of interaction modules within the luminal ER^+ ^network**. Luminal ER^+ ^interaction modules are shown projected on to the luminal ER^+ ^network. First, second and third pass nodes are indicated as coloured rectangular, octagonal and oval nodes respectively. First, second and third pass connections are indicated as thick, medium and thin red lines respectively. Solid lines indicate physical interactions, dotted lines transcriptional interactions. The different colourings of the first and second pass nodes indicate module groupings of differentially expressed genes. Third pass nodes are coloured green. Black rectangles indicate differentially expressed hubs for which no modules could be built. Click here for file

Additional file 17 **Network metrics for the luminal ER^- ^and luminal ER^+ ^module subnetworks**. The table gives the values for the parameters describing the luminal ER^- ^and luminal ER^+ ^networks both with and without the identified modules. Network manipulations were performed in Cytoscape [111], and the average network clustering <C>, average connectivity <k>, power exponent γ and mean shortest path <l> were derived with the Cytoscape Random Networks plug-in. Note that due to the highly fragmented nature of the non-module subnetwork, the mean shortest path does not constitute a reliable metric. Click here for file

Additional file 18 **List of genes common to mammary cell subpopulations and previously published datasets of estrogen-responsive genes**. The table lists estrogen-responsive genes identified in previously published datasets which were also found in the mammary epithelial cell subpopulations in the current study. Click here for file

Additional file 19 **Genes with potential roles in lineage selection/cell fate determination through paracrine signalling or as transcriptional regulators**. The table lists those genes from the three populations whose protein products have potential roles in intercellular signalling or as transcriptional regulators. *Distribution confirmed by qPCR. Click here for file

Additional file 20 ***Sox6 *over-expression maintains luminal differentiation in mammary epithelial cells *in vitro***. Additional images of immunofluorescence staining for keratin 14 (A, B) and keratin 18 (C, D) expression in primary mouse mammary epithelial cells transduced with lentivirus expressing *Sox6 *and GFP. A, C, bars = 30 μm. B, D, bars = 60 μm. Arrows in A indicate rare *Sox6*-GFP cells which are also weakly K14 positive. The majority of *Sox6*-GFP cells are K14 negative. Click here for file

Additional file 21 **Genes examined by qPCR analysis**. The table gives the gene name, symbol, TAQMAN Assays on Demand (Applied Biosystems) assay reference and Unigene ID for all qPCR probes used. Click here for file
