# Supplementary figures and images for: Transcriptome analysis of mammary epithelial subpopulations identifies novel determinants of lineage commitment and cell fate
Source: BMC Genomics. 2008 Dec 8;9:591. doi: 10.1186/1471-2164-9-591 (PMC2629782; doi:10.1186/1471-2164-9-591)

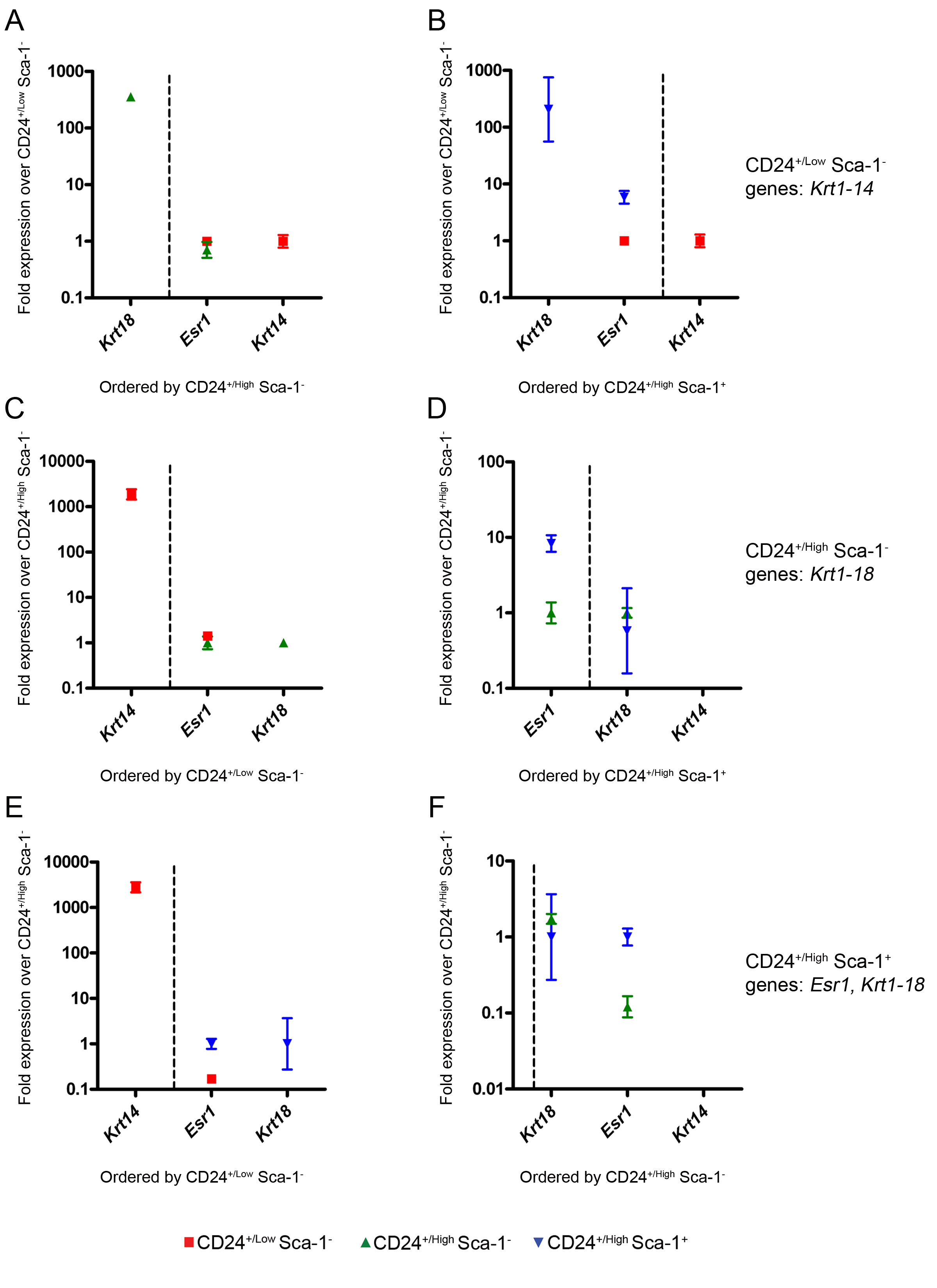

Supplement: Additional file 1 — qPCR analysis of Krt14, Krt18 and Esr1 expression in mammary epithelial subpopulations. The data describes qPCR analysis of expression of Krt14, Krt18 and Esr1 in triplicate independent samples of CD24+/Low Sca-1- cells, CD24+/High Sca-1- cells and CD24+/High Sca-1+ cells. Each data point is the mean level of expression, ± 95% confidence intervals, across the three samples of that population relative to the comparator sample. A 'round robin' comparison was used as described in the Methods. Genes considered to be characteristic of the comparator population are indicated next to each pair of graphs. Krt14 expression was undetectable in the CD24+/High Sca-1- and CD24+/High Sca-1+ cells. Krt18 expression was undetectable in the CD24+/Low Sca-1- cells. [file 1471-2164-9-591-S1.tiff]

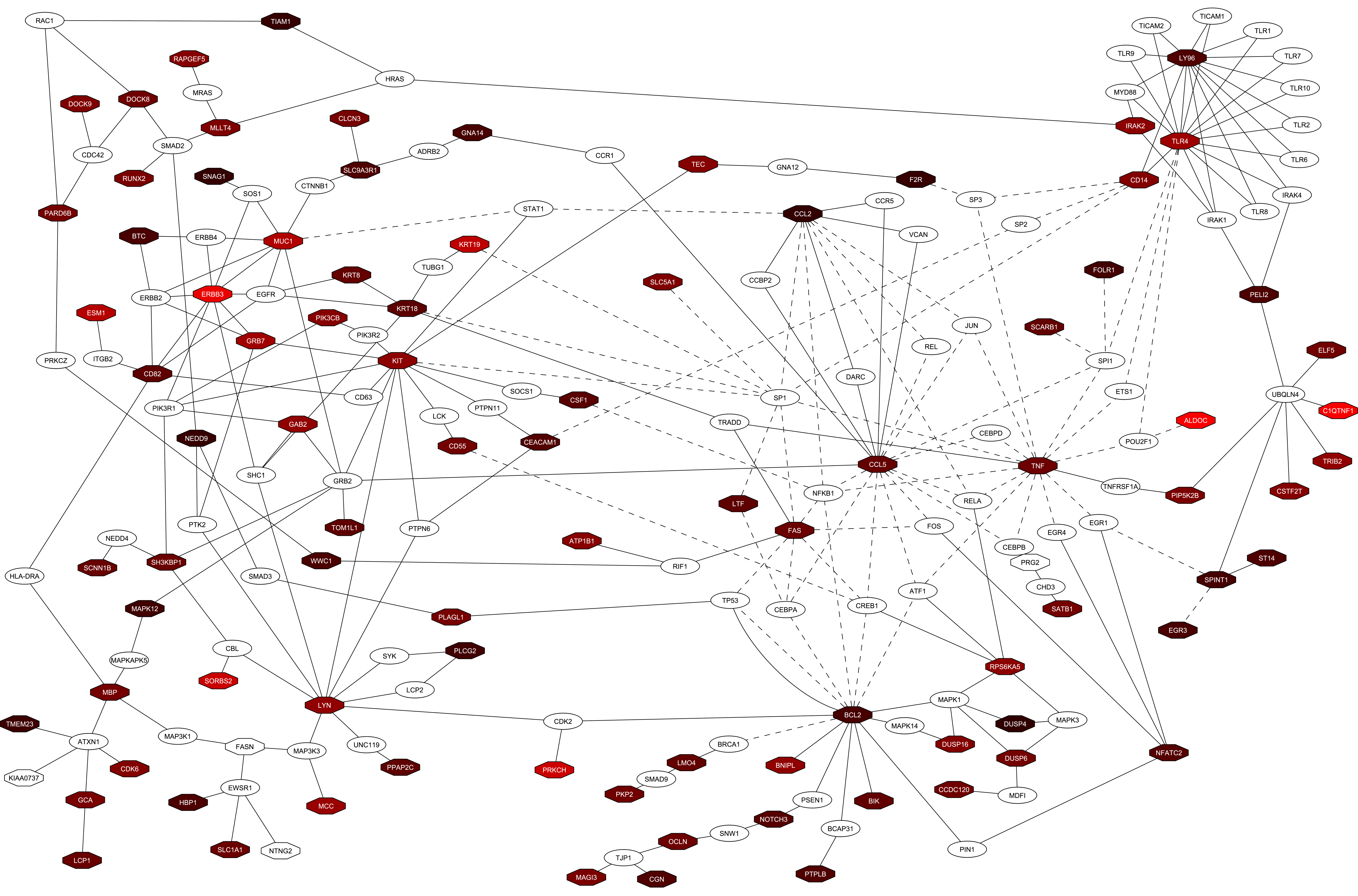

Supplement: Additional file 12 — Network interaction map for luminal ER- specific genes. Interaction data for luminal ER- specific genes based on physical interactions (solid lines) and transcriptional interactions (dashed lines). The nodes are colour coded to indicate relative strengths of expression of the gene within the cell population. Brighter reds indicate highest levels of expression. Darker reds indicate genes less strongly expressed (although still with enriched expression within the population compared to the other cell types). White nodes indicate interpolated genes used by the network mapping software to extend and link the network. [file 1471-2164-9-591-S12.tiff]

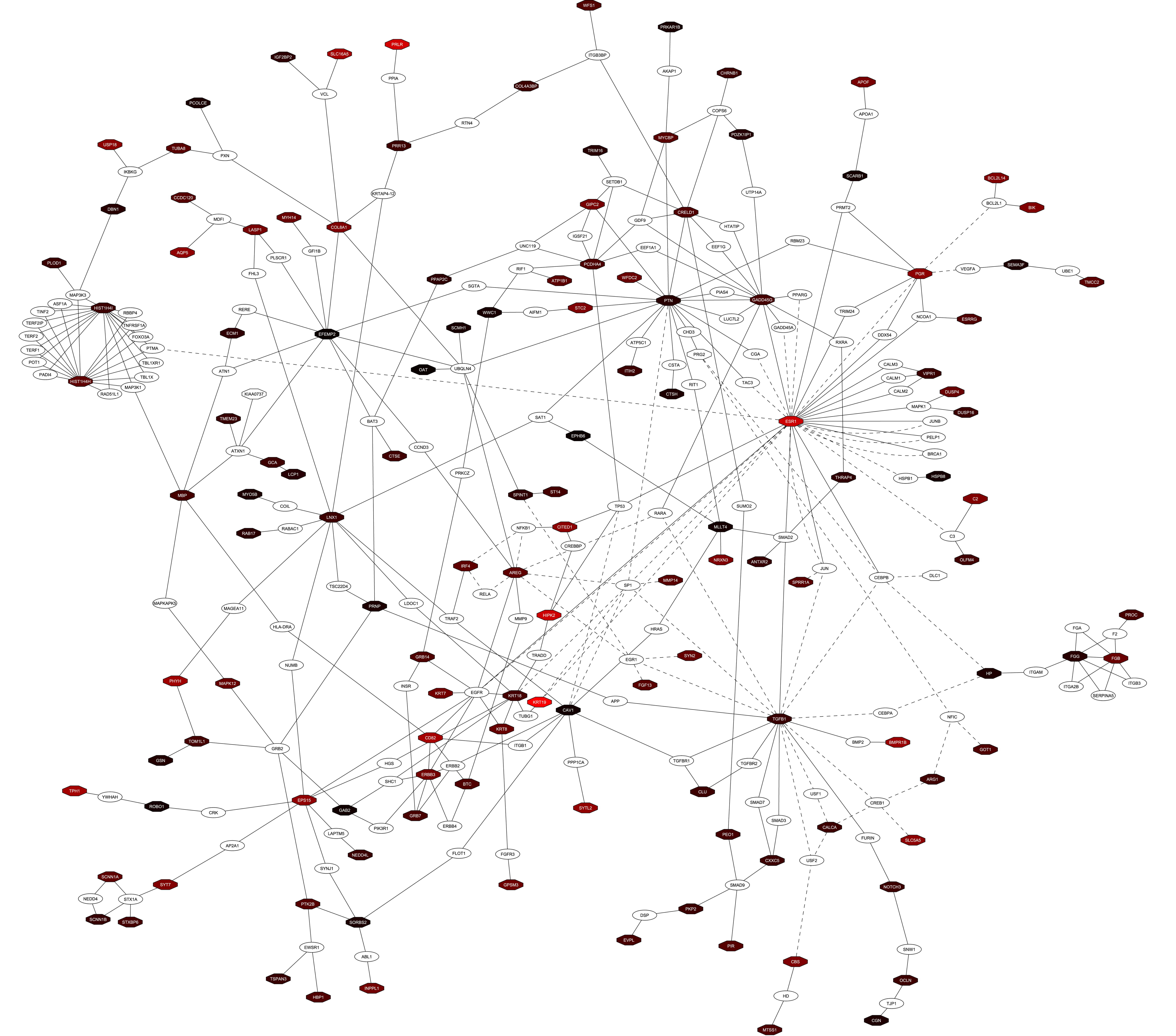

Supplement: Additional file 13 — Network interaction map for luminal ER+ specific genes. Interaction data for luminal ER+ specific genes based on physical interactions (solid lines) and transcriptional interactions (dashed lines). The nodes are colour coded to indicate relative strengths of expression of the gene within the cell population. Brighter reds indicate highest levels of expression. Darker reds indicate genes less strongly expressed (although still with enriched expression within the population compared to the other cell types). White nodes indicate interpolated genes used by the network mapping software to extend and link the network. [file 1471-2164-9-591-S13.tiff]

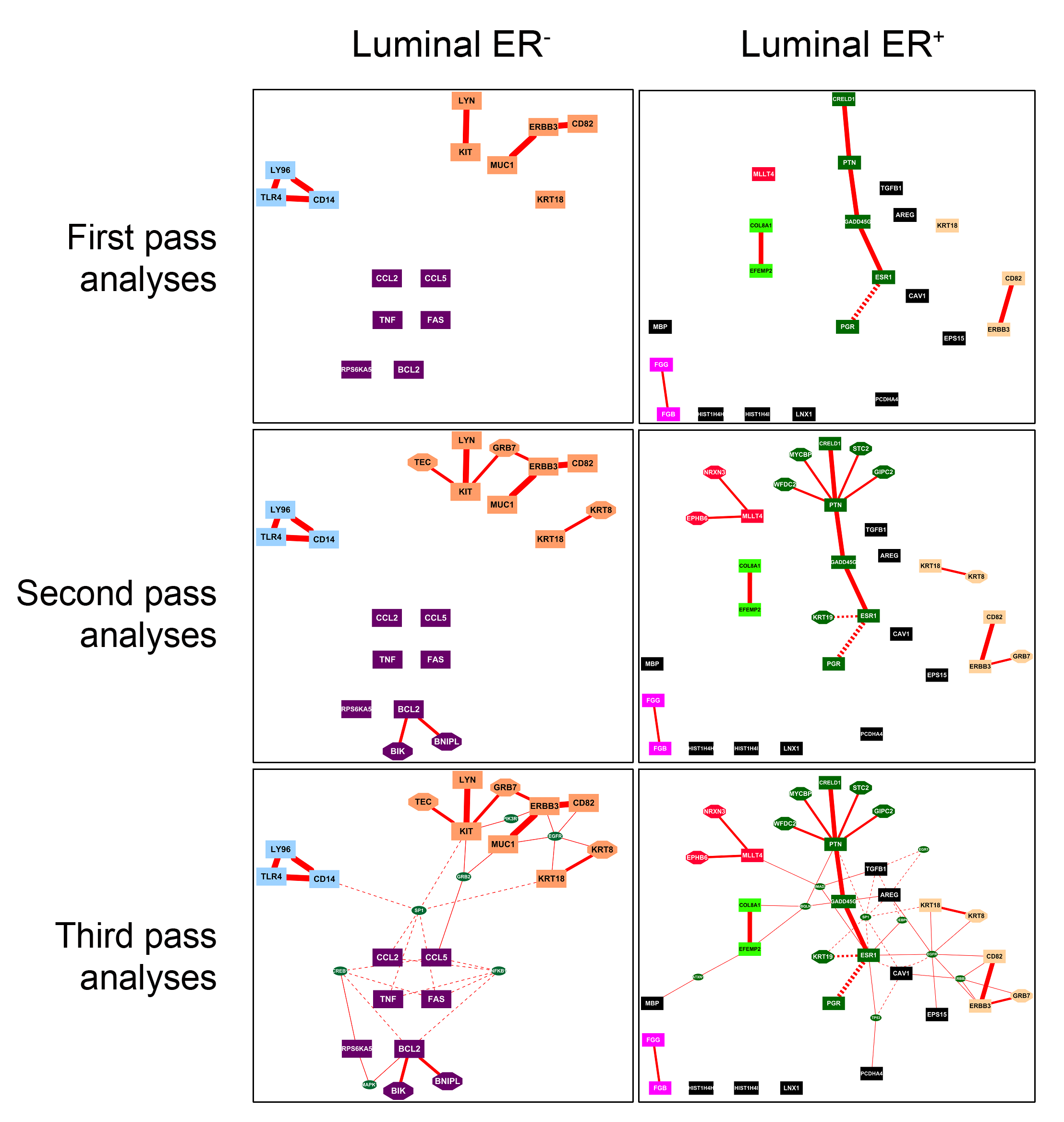

Supplement: Additional file 14 — Identification of prominent modules of differentially expressed genes in the luminal ER- and luminal ER+ networks. The results of the first, second and third pass analyses for network modules in the luminal ER- and luminal ER+ networks are shown. Rectangular nodes are first pass nodes, octagonal nodes are second pass nodes and small green oval nodes are third pass nodes. Thick red lines are first pass connections, medium size red lines are second pass connections and thin red lines are third pass connections. Black rectangles indicate differentially expressed hubs for which no modules could be built. Coloured rectangles indicate module groupings of differentially expressed genes. Solid lines indicate physical interactions, dotted lines transcriptional interactions. [file 1471-2164-9-591-S14.tiff]

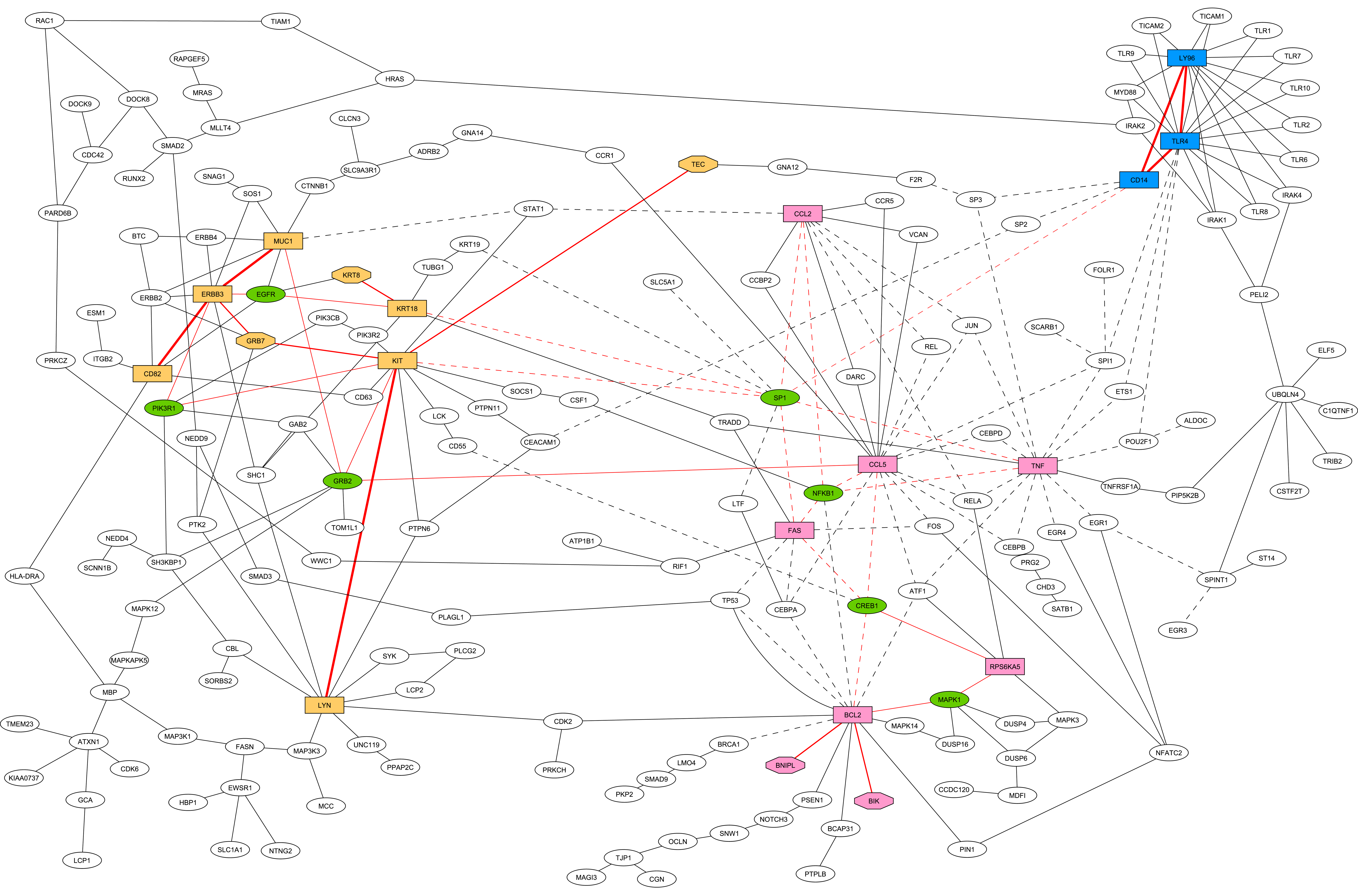

Supplement: Additional file 15 — Topology of interaction modules within the luminal ER- network. Luminal ER- interaction modules are shown projected on to the luminal ER- network. First, second and third pass nodes are indicated as coloured rectangular, octagonal and oval nodes respectively. First, second and third pass connections are indicated as thick, medium and thin red lines respectively. Solid lines indicate physical interactions, dotted lines transcriptional interactions. The different colourings of the first and second pass nodes indicate module groupings of differentially expressed genes. Third pass nodes are coloured green. [file 1471-2164-9-591-S15.tiff]

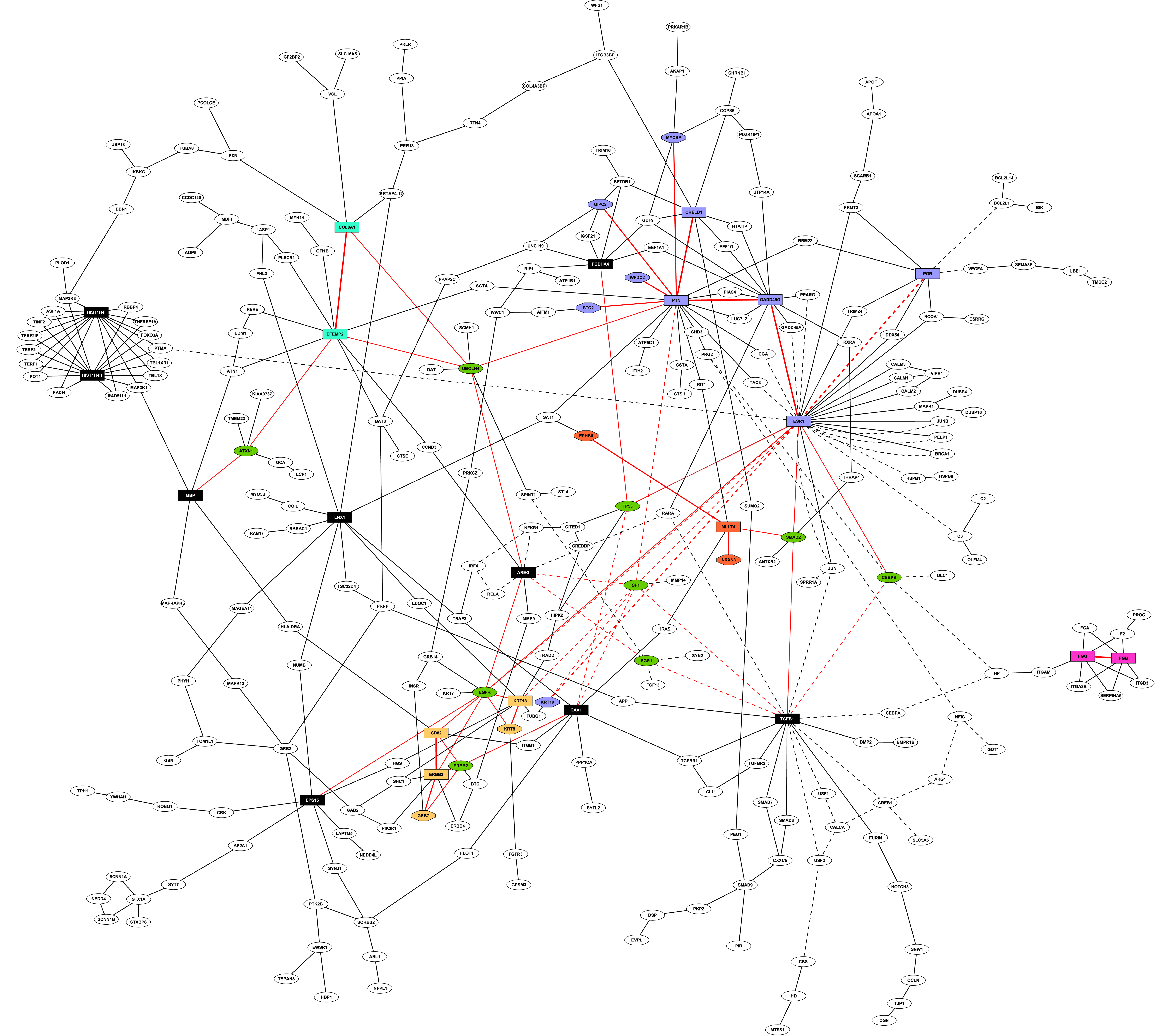

Supplement: Additional file 16 — Topology of interaction modules within the luminal ER+ network. Luminal ER+ interaction modules are shown projected on to the luminal ER+ network. First, second and third pass nodes are indicated as coloured rectangular, octagonal and oval nodes respectively. First, second and third pass connections are indicated as thick, medium and thin red lines respectively. Solid lines indicate physical interactions, dotted lines transcriptional interactions. The different colourings of the first and second pass nodes indicate module groupings of differentially expressed genes. Third pass nodes are coloured green. Black rectangles indicate differentially expressed hubs for which no modules could be built. [file 1471-2164-9-591-S16.tiff]

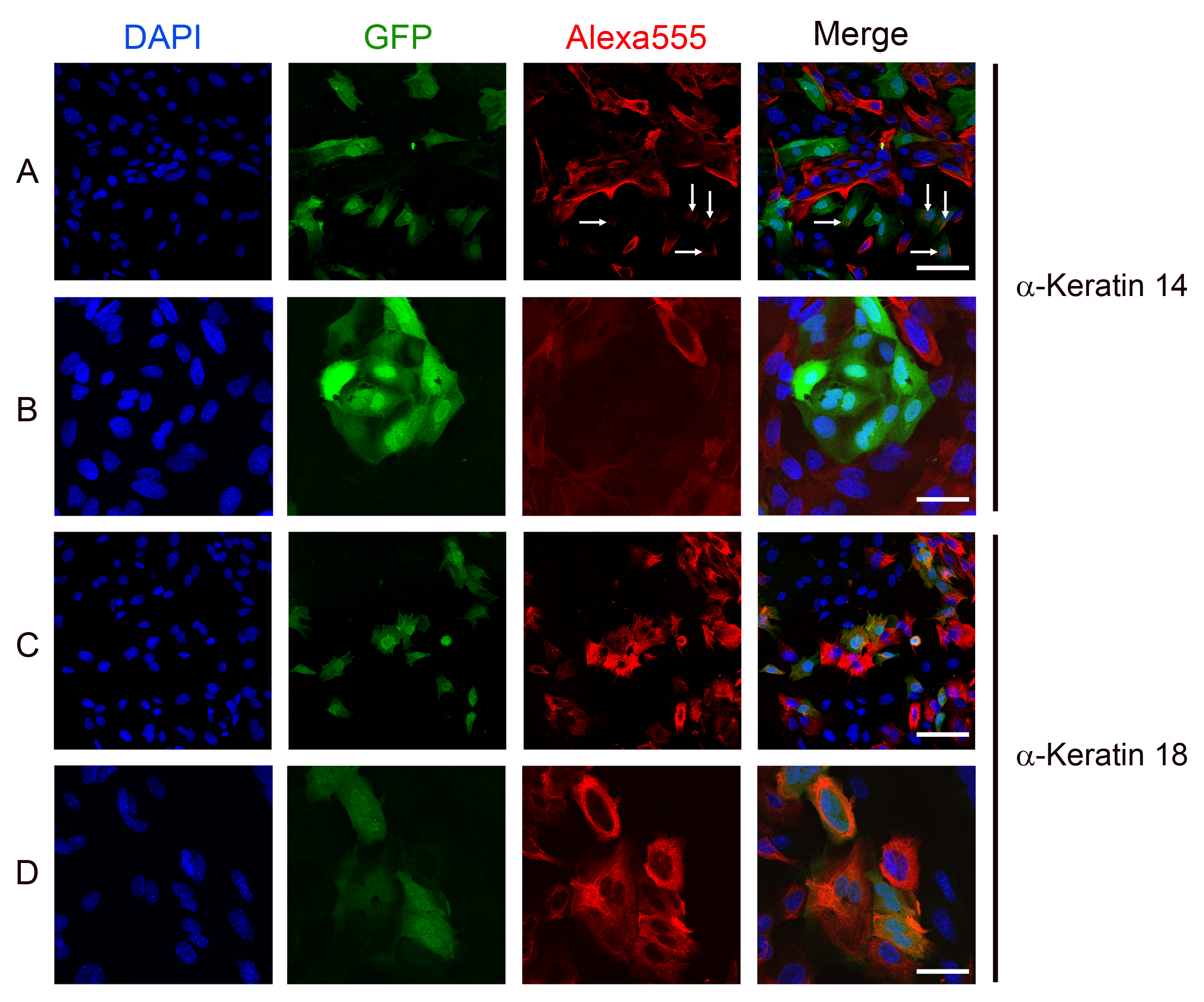

Supplement: Additional file 20 — Sox6 over-expression maintains luminal differentiation in mammary epithelial cells in vitro. Additional images of immunofluorescence staining for keratin 14 (A, B) and keratin 18 (C, D) expression in primary mouse mammary epithelial cells transduced with lentivirus expressing Sox6 and GFP. A, C, bars = 30 μm. B, D, bars = 60 μm. Arrows in A indicate rare Sox6-GFP cells which are also weakly K14 positive. The majority of Sox6-GFP cells are K14 negative. [file 1471-2164-9-591-S20.tiff]
